# Analysis of the Structural Mechanism of ATP Inhibition at the AAA1 Subunit of Cytoplasmic Dynein-1 Using a Chemical “Toolkit”

**DOI:** 10.3390/ijms22147704

**Published:** 2021-07-19

**Authors:** Sayi’Mone Tati, Laleh Alisaraie

**Affiliations:** School of Pharmacy, Memorial University of Newfoundland, 300 Prince Philip Dr, St. John’s, NL A1B 3V6, Canada; msmtp4@mun.ca

**Keywords:** dynein motor domain, ATP hydrolysis, inhibition, ciliobrevin, dynapyrazole, analogues

## Abstract

Dynein is a ~1.2 MDa cytoskeletal motor protein that carries organelles via retrograde transport in eukaryotic cells. The motor protein belongs to the ATPase family of proteins associated with diverse cellular activities and plays a critical role in transporting cargoes to the minus end of the microtubules. The motor domain of dynein possesses a hexameric head, where ATP hydrolysis occurs. The presented work analyzes the structure–activity relationship (SAR) of dynapyrazole A and B, as well as ciliobrevin A and D, in their various protonated states and their 46 analogues for their binding in the AAA1 subunit, the leading ATP hydrolytic site of the motor domain. This study exploits in silico methods to look at the analogues’ effects on the functionally essential subsites of the motor domain of dynein 1, since no similar experimental structural data are available. Ciliobrevin and its analogues bind to the ATP motifs of the AAA1, namely, the walker-A (W-A) or P-loop, the walker-B (W-B), and the sensor I and II. Ciliobrevin A shows a better binding affinity than its D analogue. Although the double bond in ciliobrevin A and D was expected to decrease the ligand potency, they show a better affinity to the AAA1 binding site than dynapyrazole A and B, lacking the bond. In addition, protonation of the nitrogen atom in ciliobrevin A and D, as well as dynapyrazole A and B, at the N9 site of ciliobrevin and the N7 of the latter increased their binding affinity. Exploring ciliobrevin A geometrical configuration suggests the *E* isomer has a superior binding profile over the *Z* due to binding at the critical ATP motifs. Utilizing the refined structure of the motor domain obtained through protein conformational search in this study exhibits that Arg1852 of the yeast cytoplasmic dynein could involve in the “glutamate switch” mechanism in cytoplasmic dynein 1 in lieu of the conserved Asn in AAA+ protein family.

## 1. Introduction

Motor proteins, dynein, kinesin, and myosin, in eukaryotic cells are responsible for transporting cargoes within cells [1]. Dynein and kinesin perform their function in conjunction with the cytoskeletal protein microtubule (MT) [1]. MTs comprise 11–16 protofilament biopolymers [2] consisting of αβ-heterodimer proteins [1,3]. Nine subfamilies compose the dynein family, namely, seven axonemal and two cytoplasmic, dynein 1 and dynein 2 [4]. Cytoplasmic dynein 1 drives retrograde axonal transport [5] and also plays a role in the mitosis process of cell division [1]. Cytoplasmic dynein 2 guarantees transportation of cargoes through MTs in flagella, as well as motile and primary cilia [6,7]. This isoform is also referred to as intraflagellar transport (IFT) dynein [6,7]. IFT is critical to the Hedgehog pathway (Hh pathway), which is an essential mediator during the development of the embryo and oncogenesis [8]. It facilitates anterograde and retrograde trafficking of transcription factors such as Gli1 and Gli2 during the Hh pathway [9,10]. Impairment of dynein 2 could disturb the Hh pathway since it is involved in IFT [6,7]. Inhibitors of dynein such as ciliobrevin analogues cause the inhibition of the Hh pathway [10]. Malfunction of dynein can promote cancer cell proliferation [7], as dynein 2 is involved in the Hh pathway and oncogenesis process [6].

Defects in the heavy chain of dynein are associated with neurodegenerative diseases (NDDs) [11], characterized by the degradation of neurons. NDDs refer to an array of neurological disorders, including Parkinson’s disease (PD), Huntington’s disease (HD), Alzheimer’s disease (AD), and motor neuron diseases [5]. Three common features observed in the NDDs are the presence of protein aggregates, the involvement of nonautonomous factors, and the dysfunction in axonal transport [5]. PD is characterized by the death of dopaminergic cell groups producing dopamine in the substantia nigra, which results in symptoms such as resting tremors, bradykinesia, and rigidity of limbs [5]. HD is a condition associated with disturbance in muscle coordination and cognitive impairment caused by a polyglutamate fragment on the huntingtin protein resulting from the repetition of the CAG codon in exon 1 of the gene responsible for the mentioned protein [5,11]. Both PD and HD affect basal ganglia in the brain [5]. The occurrence of axonal dystrophy in the brain of patients with PD indicates abnormalities in axonal transport. Dysfunction of the axonal transport, observed in animal and cellular models, represents indirect evidence of dynein involvement in PD and HD pathologies. Dysfunction of dynein causes the Golgi apparatus to fragmentize, a phenomenon observed in the brain of patients with PD, as well as cellular and animal models of PD and HD [5]. AD, affecting 25 million individuals globally, is characterized by progressive deterioration of memory that results in this pathology and the loss of cognitive abilities, poor judgment, and speech impairment [11]. AD is marked by the presence of clusters of misfolded proteins, i.e., amyloid plaques consisting primarily of amyloid β peptides (Aβ) in the brain of patients with AD [5]. Indirect evidence obtained through the knockdown of dynein, causing an increase in Aβ peptides, suggested the involvement of dynein in AD; however, further experiments are needed to exhibit a direct correlation between dynein activity and AD [5]. Eyre et al. revealed that dynein plays an essential role in the transportation of NS5A, a hepatitis C viral protein, inside cells. Dynein ensures the efficient replication of the virus, as well as the assembly of virions [6].

**Figure 1:**
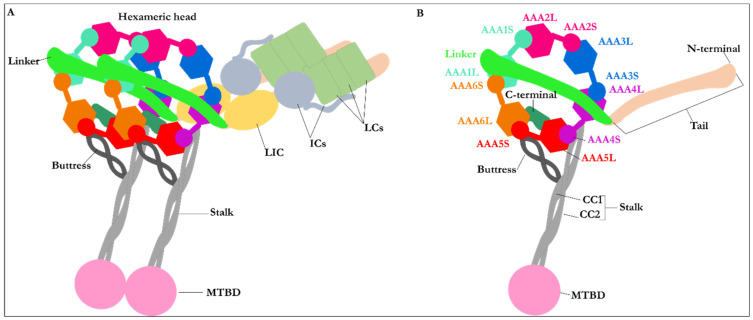
The multidomain structure of cytoplasmic dynein. (**A**) Schematic representation of the homodimer cytoplasmic dynein. For simplicity, only one monomer is labeled. The homodimer represents two heavy chains, two LICs, four ICs, and six LCs. Each set of the two heavy chains consists of a tail, a linker, a hexameric head, a buttress, a stalk, and an MTBD. The figures (e.g., the LCs, ICs, and LICs) are schematic. They do not represent their actual shape, (**B**) Schematic representation of the heavy chain of cytoplasmic dynein in its post-powerstroke conformation, with the linker straight and positioned on AAA4 near the stalk. The hexameric head represents the six AAA+ subunits with small (AAA + S) and large (AAA + L) subunits.

Dynein was discovered before kinesin [4]; however, the former has been more challenging than the latter to solve its three-dimensional structure and to understand the exact mechanism of action of its multidomain construction. Indeed, the complexities result from its massive size, its two heavy chains of each 530 kDa [7]. Despite the complexity of dynein structure, characterization of some of its substructures or domains utilizing X-ray crystallography, electron microscopy, and mutagenesis studies have provided insights into understanding its function and role in the cell [7]. Dynein is a homodimer protein [1], composed of two heavy chains (HCs), each 530 kDa, two light intermediate chains (LICs), each 74 kDa, four intermediate chains (IC) with weight varying between 53 kDa and 59 kDa each, and six light chains (LCs), each 10–14 kDa [11]. The heavy chain of dynein consists of the tail, the linker, the hexameric head, the buttress, the stalk domains, and the microtubule-binding domain (MTBD) [12,13]. The N-terminus of dynein, representing approximately one-third of the 530 kDa heavy chain, constitutes the tail and the linker [1,14] (Figure 1).

The tail of the heavy chain of dynein represents the site for cargo binding, where the dimerization of both monomers occurs [1]. The tail also binds to the LICs and ICs [1,5]. The linker domain follows the tail and is thought to be involved in a force-generating process as its position changes upon binding of ATP, resulting in the motility of dynein [1]. The stalk of ~10–15 nm in length is attached to the MTBD at the C-terminus [15]. It is linked to and supported by the buttress [1] (Figure 1A).

**Figure 2:**
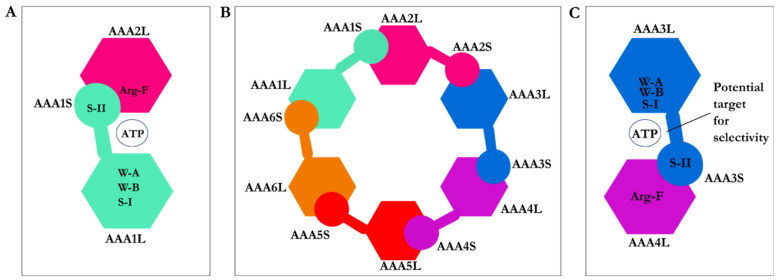
Composition of the AAA+ subdomains. (**A**) The AAA1 nucleotide-binding site composed of the small (AAA1S) and large (AAA1L) subunits of AAA1 and the prominent (AAA2L) subunit of AAA2. ATP motifs are represented: walker-A (W-A), walker-B (W-B), sensor I (the S-I), sensor II (the S-II) in AAA1, and Arginine finger (Arg-F) in AAA2, (**B**) Hexameric head of cytoplasmic dynein, (**C**) The AAA3 nucleotide-binding site composed of its small (AAA3S) and large (AAA3L) subunits and the large subunit of AAA4. ATP motifs are represented as the walker-A (W-A), the walker-B (W-B), the sensor I (the S-I), the sensor II (the S-II) in AAA3, and the arginine finger (Arg-F) in AAA4.

The head or motor domain of dynein is comprised of six AAA+ subunits, four of which (AAA1 to AAA4) possess a nucleotide-binding site at the interface between one subdomain and the subsequent subdomain; the AAA1 nucleotide-binding site is enclosed between the AAA1 and AAA2 subdomains [1,12]. Three of the four binding sites, AAA1, AAA3, and AAA4, present the ability to hydrolyze ATP [1,12]. Each of the six AAA+ subdomains encompasses a small and a large subunit linked by a flexible unfolded segment [1] (Figure 1B and Figure 2B).

**Figure 3:**
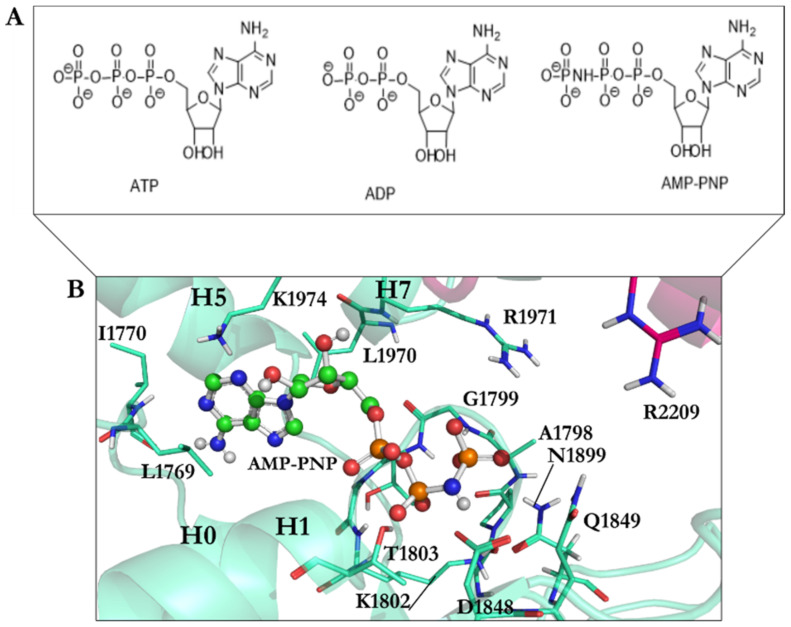
(**A**) The chemical structures of ATP, ADP, and AMPPNP; (**B**) AMPPNP in the ball-and-stick representation (green) interacts with amino acids in the AAA1 binding site of cytoplasmic dynein 1. Color code: AAA1 in cyan blue and AAA2 in sharp pink.

AAA1 is the primary site of ATP hydrolysis in cytoplasmic dynein [1] since hydrolysis of ATP at this site is critical for dynein motility [16] and conserved in the dynein family [1]. AAA3 is the second major site of ATP hydrolysis [1], as mutation of K2675T in the *D. discoideum* species reduced ATPase activity of dynein by approximately 20-fold [16]. The nucleotide-binding sites of cytoplasmic dynein, similar to other AAA+ family members, display the following ATP motifs: the walker-A (GXXXGK) or P-loop, the walker-B (catalytic Asp and Glu), the S-I (Asn), the S-II (Arg), an arginine finger (Arg), and directly interacting amino acids with the nucleotide base [1,7] (Figure 2 and Figure 3).

Considering the vital role of the heavy chain defects in causing some of the significant NDDs [11] and the gigantic size of dynein (~1.2 MDa), small-molecule inhibitors are suitable means to examine how the function of the dynein motor domain could be regulated or inhibited. Therefore, this structure–activity relationship (SAR) study attempted to elucidate the structural effect of ciliobrevin A and D, as well as their analogues, on their potential regulatory or inhibitory [6] mechanisms concerning the function of the motor domain in cytoplasmic dynein 1. As the size and the complexity of the various structural domains of dynein lead to considerable challenges in solving their atomistic holo or apo structure in vitro, in silico methods in the presented work were utilized to address some of the current shortcomings.

## 2. Materials and Methods

### 2.1. Structure of Dynein Motor Subdomains

Three crystal structures of the motor domain of cytoplasmic dynein 1, including the linker, are available in the Protein Data Bank (PDB) [17]. The three crystal structures studied here are motor domains of *Dictyostelium* motor ADP (3VKG) [18] from *Dictyostelium discoideum*, as well as yeast motor apo (4AKG) [19] and yeast motor AMPPNP (4W8F) [20], both from *Saccharomyces cerevisiae*. The crystal structures were selected on the basis of their resolution, the conformation of the AAA1 binding site, and the nucleotide substrate in the binding site. Due to the complexity of the cytoplasmic dynein structure, the resolution of the crystal structures is low, as it ranges from 2.41 Å (*Dictyostelium* motor ADP (3VKG) [18]) to 3.54 Å (yeast motor AMPPNP (4W8F) [20]); however, they are the highest-quality structures of the domain currently available from the Protein Data Bank (Table 1).

The hexameric head from the *Dictyostelium* motor ADP [18] crystal structure accommodates one ADP molecule in the AAA1, AAA2, AAA3, and AAA4 subunits (3VKG) [18]. The hexameric head from the yeast motor AMPPNP crystal structure (4W8F) [20] possesses an AMPPNP molecule in each of the subunits. The yeast motor apo crystal structure (4AKG) [19] presents the AAA1 binding site in its unliganded state, whereas an ATP is found in the AAA2 and an ADP in the AAA3 binding site (4AKG) [19]. The three crystal structures have their linker in the post-powerstroke conformation. The linker is straight in the *Dictyostelium* motor ADP [18] crystal structure (3VKG) [18], spanning the AAA1 to AAA5 subunits. In comparison, the linker stretches from AAA1 to AAA4 in the yeast motor AMPPNP (4W8F) [20] and the yeast motor apo (4AKG) [19] crystal structures. The AAA1 binding sites of yeast motor AMPPNP [20] and *Dictyostelium* motor ADP [18] are in holo states in their crystal structures, with AMPPNP and ADP bound in each, respectively (Table 1).

### 2.2. Protein Structure Preparation

The accession codes of the three-dimensional structure of the motor domain of dynein collected from the PDB platform are 4AKG [19] from *S. cerevisiae* (Uniprot P36022 [21]), 4W8F [20] from *S. cerevisiae* (Uniprot P36022 [21]), and 3VKG [18] (Uniprot P34036 [21]) from *Dictyostelium Discoideum* (Uniprot P34036 [21]) (Table 1).

The amino-acid sequences of the two species, *S. cerevisiae* (P36022) and *D. discoideum* (P34036), were aligned according to the ClustalW algorithm [22,23]. There is a sequence identity of ~25% between the cytoplasmic dynein of *S. cerevisiae* (P36022) containing 4092 residues and *D. Discoideum* (P34036) containing 4730 residues. According to the sequence alignment (Tyr1758–Val2273: *S. cerevisiae* and Tyr1936–Leu2531: *D. discoideum*), there are 35% conserved residues within the AAA1 and AAA2 subdomains of both species (Figure S1 and Table 2).

The yeast motor AMPPNP crystal structure (4W8F) [20] was subjected to E1849Q mutation to prevent ATP hydrolysis at the AAA1 nucleotide-binding site [20]. The yeast motor AMPPNP crystal structure was considered suitable for docking ATP competitive inhibitors in the AAA1 nucleotide-binding site and corresponded to the conformation of dynein before ATP hydrolysis [20]. The *Dictyostelium* motor ADP (3VKG) possesses a molecule of ADP in the AAA1 nucleotide-binding site corresponding to the configuration succeeding ATP hydrolysis [18]. In contrast, the yeast motor apo (4AKG) pertains to motor domain conformation with low-affinity nucleotides binding [19]. Thus, the yeast motor AMPPNP conformation (4W8F) [20] was chosen over the *Dictyostelium* motor ADP (3VKG) [18] or the yeast motor apo (4AKG) [19] for the ligand docking experiment.

The missing residues (i.e., crystallographically unsolved) from the motor chain A in 4W8F [20] (i.e., Ala2025–Leu2029, Lys2950–Val2953, and Lys3659–Arg3668) were modeled and completed on the basis of the primary structure of the cytoplasmic dynein heavy chain of *S. cerevisiae* (P36022) (Table 1).

Fourteen residues from AAA1 and AAA2 subunits were located in the nucleotide-binding site. They consisted of the W-A or the P-loop region GPAGTGKT [4,7,18] (Gly1796–Thr1803 in *S. cerevisiae* and Gly1974–Thr1980 in *D. discoideum*), the W-B region [4,7,18] (Asp1848 and Glu1849 in *S. cerevisiae* compared to Asp2026 and Glu2027 in *D. discoideum)*, the S-I [4,7,18] (Asn1899 in *S. cerevisiae* and Asn2078 in *D. discoideum*), the S-II (Arg1971 in *S. cerevisiae* and Arg2150 in *D. discoideum)*, the Arg finger (Arg2209 in *S. cerevisiae* and Arg2410 in *D. discoideum)* [4,7,18], and the N-loop [4,7,18] (Leu1769 and Ile1770 in *S. cerevisiae* compared to Leu1947 and Val1948 in *D. discoideum*) (Table 3).

The retrieved X-ray crystal structure (4W8F) [20] was truncated to keep the required domains potentially affecting the nucleotide-binding sites to reduce the necessary CPU time for the motor subdomain conformational search. That reduced the number of atoms for calculating bonding and nonbonding interactions among ligand and protein atoms. The resulting truncated structure included the dynein hexameric head (AAA1–AAA4: Tyr1758–Val2984 and AAA5–AAA6: Leu3370–Asn3970), the linker subunit (within the tail: Gly1363–Gln1757), and a part of the stalk (Ile2993–Ser3125) interacting with the hexameric head. GROMACS [24] package (v. 2016.5, University of Groningen Royal Institute of Technology, Groningen, The Netherlands & Uppsala, Sweden) with the Gromos 96 force field 54A7 [25], was utilized for generating topology of protein atoms and energy minimization in vacuo to optimize bond lengths, angles, and orientation of the residues in the protein structure before docking any ligands.

### 2.3. Ligands 3D Structure Preparation 

The AMPPNP’s atomic coordination at the AAA1 site (4W8F) [20] was used as the reference. The binding site region was specified at a 15.0 Å radius spherical region around the reference structure as the center, covering an extra 2.0 Å broader region than that occupied by the AMPPNP interacting amino acids in the binding site of the AAA1 domain of cytoplasmic dynein 1 (Figure 3).

**Figure 4:**
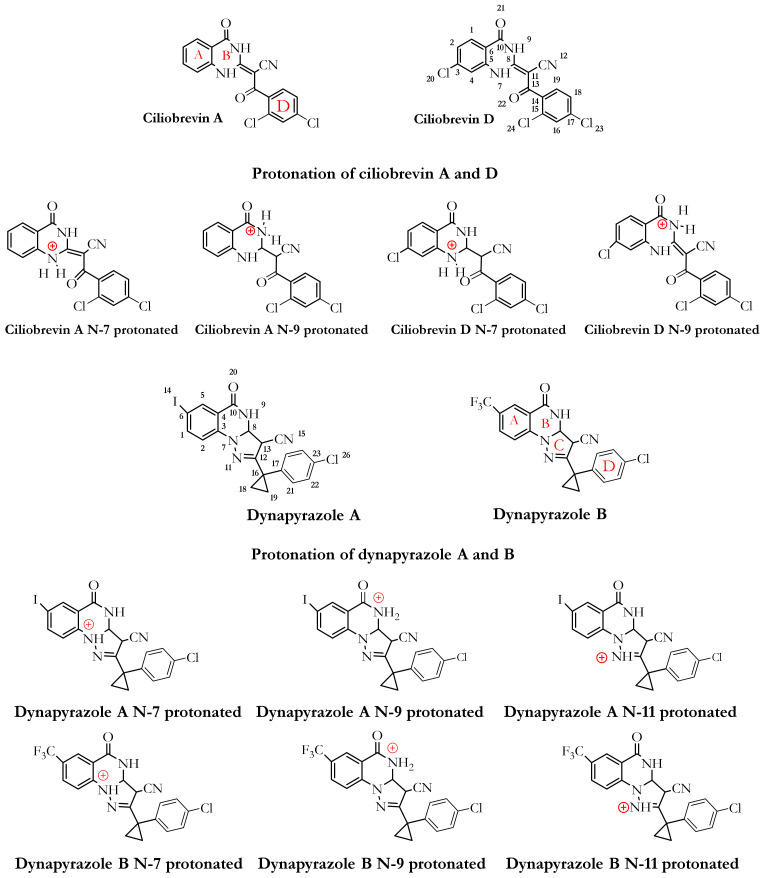
Chemical structures of ciliobrevin A and D, dynapyrazole A and B, and their protonated structures in the ligand library. Ciliobrevin A and D are analogues 1 and 2, respectively. Chemical structures and atom numbering were obtained utilizing Chemdraw software (v.19.1).

**Figure 5:**
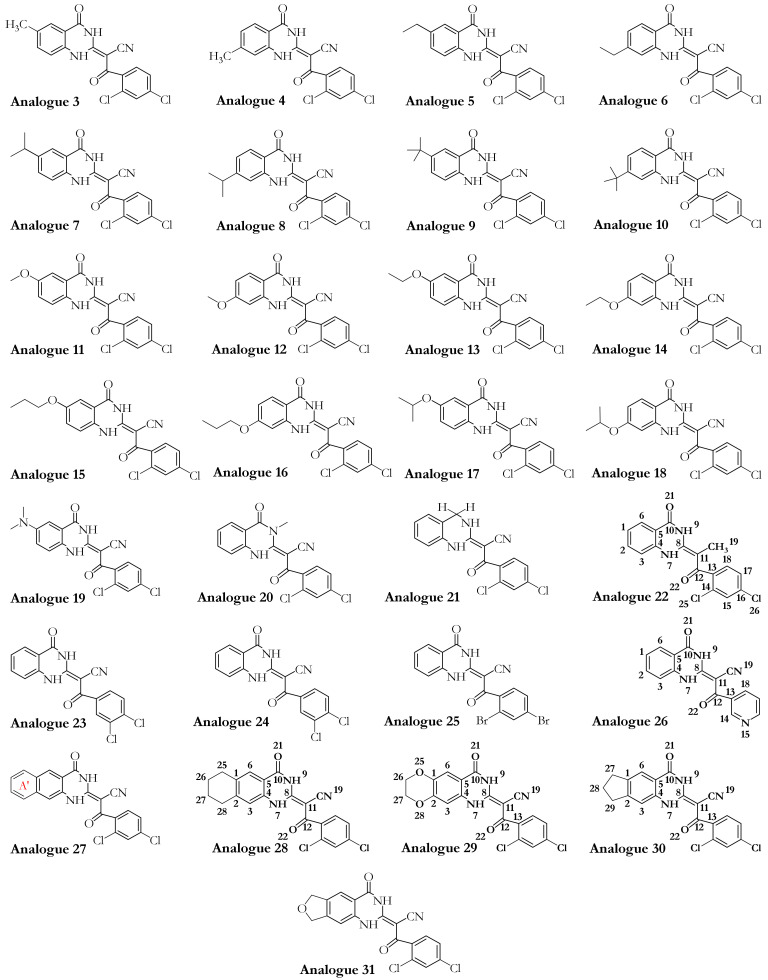

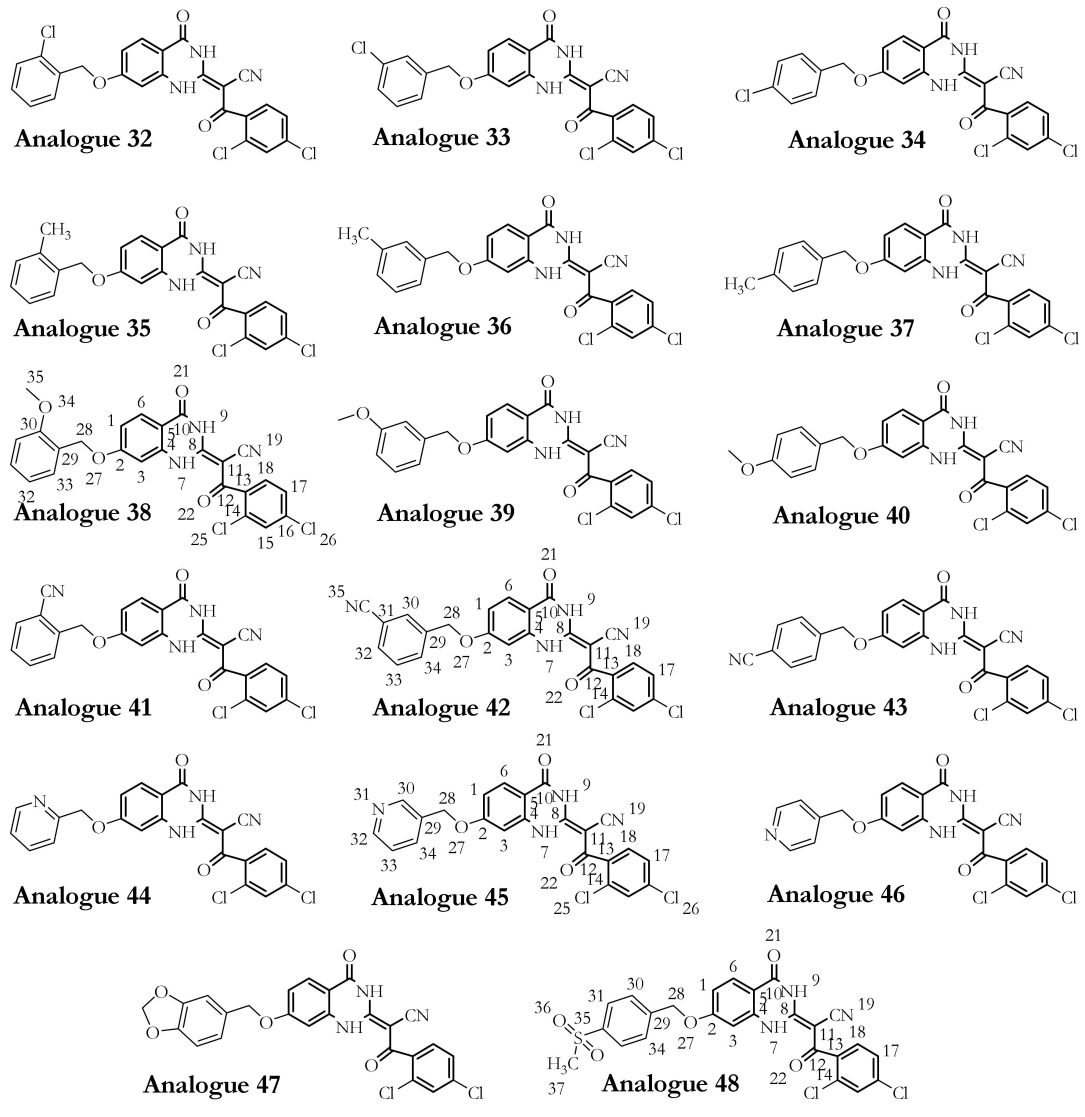
Forty-six analogues (from analogues 3–48) of ciliobrevin in the ligand library. Chemical structures and atom numbering were obtained utilizing Chemdraw software (v.19.1).

A library of 63 ligands (i.e., a chemical toolkit in this study) was created using SYBYL-X 2.1.1 (Certara Corporation©, St. Louis, MO, USA). Three-dimensional structures of the ligands were built up individually and minimized stepwise using the steepest descent algorithm according to the Tripos force field, with 0.0001 kJ/mol energy gradient and 10,000,000 iterations. The library contained previously synthesized and in vitro studied 46 analogues of ciliobrevin [10] and dynapyrazole A and B [26], as well as the protonated forms of the lead compounds ciliobrevin A and D and dynapyrazole A and B modeled in silico. It also included the nucleotides ATP, ADP, and AMPPNP, a nonhydrolyzable analogue of ATP (Figure 3, Figure 4 and Figure 5).

ATP and ADP are the endogenous substrates of dynein 1 [1]. Since the crystal structure of yeast motor AMPPNP did not possess ATP or ADP in any of the four nucleotide-binding sites (AAA1–AAA4), the endogenous substrates were docked into the AAA1 binding site to study their binding mode and quantify the magnitude of their binding affinity versus that of each ligand in the library. The deprotonated forms of ATP, ADP and AMPPNP, were based on the ATP pKa values [27]. The pH was 8.0 during the crystallization of the yeast motor AMPPNP(4W8F) [20], and the pKa of γ-phosphate is approximately 6.49 [27]. In comparison, the pKa of the α- and β-phosphates of ATP is estimated at ~1.6 [27]. Therefore, ATP, ADP, and AMPPNP molecules were also built and assessed in their fully deprotonated state and subjected to energy minimization. The protonated ciliobrevin A and D and dynapyrazole A and B structures were built up and energetically minimized. The pKa of the inhibitors has not yet been experimentally defined. A study on the different components of the ligands’ chemical structures helped to study the effect of the most probable protonation states on their binding affinity. The pKa of arylamine groups, existing in the structures of the ligands, varies between 9–10 [28], meaning that, at the physiological pH, an arylamine (i.e., consisting of the N9 atom of dynapyrazole A and B, ciliobrevin A and D, and their analogues) could be protonated. It is noteworthy that the lone-pair electrons of the N7, N9, and N11 in dynapyrazole A and B could be involved in delocalized electronic systems of A, B, and C fragments, reducing the availability of the lone-pair electrons for protonation. Furthermore, the pKa of the quinazoline-4(3*H*)-one moiety of dynapyrazole (i.e., ring A and B) is expected to be more acidic than the estimated 3.51 of quinazoline [29], due to the electron withdrawal effect of the oxygen. Thus, the moiety is more likely to be deprotonated at the physiological pH (Figure 4).

FlexX [30,31] docking software, embedded in the LeadIT software package (v.2.1.8, BioSolveIT, Sankt. Augustin, Germany), was utilized for ligand–protein binding mode predictions, energy estimation, and ranking the solutions. It predicts the ligand–protein interactions on the basis of the incremental construction algorithm [32]. There are three fundamental stages to the FlexX docking algorithm: selecting a base fragment, placing the base fragments into the active site, and incrementally constructing the complex, followed by calculating the interaction energies according to the Böhm scoring function for ranking the docking solutions [33,34].

## 3. Results and Discussion

### 3.1. Dynapyrazole, Ciliobrevin, and Their Analogues

In vitro and in vivo studies of ciliobrevin A and D, the two ATP-competitive ligands, have shown that they nonselectively bind to the ATP-binding sites of the hexameric head of both cytoplasmic dynein 1 and dynein 2 [7,10]. Dynapyrazole A and B resulted from a chemical structure modification to produce ciliobrevin analogues with higher potency [26] to overcome geometric isomerization complexity caused by the C8–C11 double bond in ciliobrevin (Figure 4).

Unlike the ciliobrevin analogues, which abrogate both MT-stimulated and basal ATPase activity, dynapyrazole analogues inhibit MT-stimulated ATPase activity with high potency without affecting basal ATPase activity [26]. This feature resembles She1, a microtubule-associated protein (MAP) that effectively reduces MT-stimulated ATPase activity without significantly decreasing its basal activity [35]. Experiments have shown that ciliobrevin A and D, which bind to AAA1, might bind to the AAA3 site [10]. In contrast, analogues of dynapyrazole, especially compound 20, abolished basal dynein activity by binding to AAA3 and AAA4 [36]. Forty-six analogues of ciliobrevin A and D were proposed to have potentially higher selectivity and potency than ciliobrevin A against dynein 2 [7]. However, only the IC_50_ values of four analogues (i.e., 18, 37, 43, and 47) against dynein 1 and 2 were reported [7] (Figure 5 and Table 4).

### 3.2. Binding Studies of Dynapyrazole, Ciliobrevin, and Their Analogues

**Figure 6:**
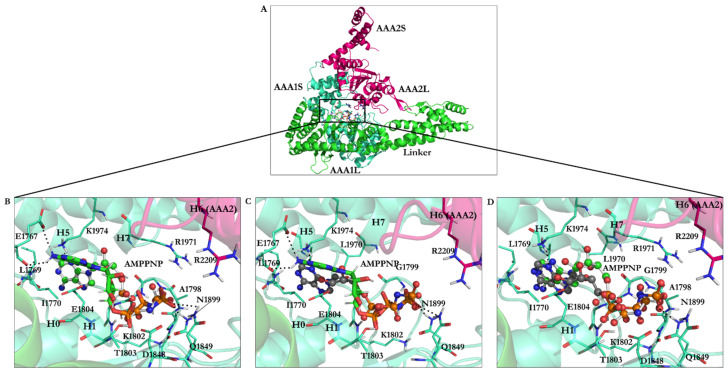
AMPPNP in the AAA1 binding site of dynein. (**A**) The linker and the AAA1 and AAA2 subunits of dynein and illustration of their inter-subunit binding site. (**B**) Docking solution of the minimized AMPPNP in the AAA1 binding site of the minimized conformation from the yeast motor AMPPNP crystal structure (4W8F). The docking solution is in stick representation, while the crystal structure of AMPPNP is in ball-and-stick representation. (**C**) The energy-minimized AMPPNP in the AAA1 binding site of the energy-minimized structure of yeast motor AMPPNP (4W8F) superimposed with the docking solution of the reference ligand, AMPPNP, from the crystal structure. (**D**) Crystal structure of AMPPNP docked in the AAA1 binding site of the crystal structure of yeast motor AMPPNP (4W8F). The calculated conformations (black-gray) ball-and-stick representations and the crystal structure of AMPPNP (reference, green ball-and-stick representation). Nonessential hydrogen atoms are not shown for simplicity.

Docking of the AMPPNP, obtained from the crystal structure, into the binding site of the yeast motor AMPPNP [20] of dynein resulted in a conformation with the lowest RMSD (1.67 Å) and binding energy (−22.06 kJ/mol). The ligand interacted with the N-loop (Pro1766–Leu1774) via residues Leu1769 and Ile1770, the W-A region (Gly1799–Thr1803), the β6 strand including the S-I motif (Ala1893–Asn1899) via Asn1899, Ile1929 from H5 (Ser1926–Ile1936), and Leu1970 and Lys1974 from the H7 (Leu1970–Pro1982) (Figure 6C,D, Figure S1, and Table 5).

Superposition of the domain crystal structures showed that the W-A region (Gly1796–Thr1803) in the yeast motor apo (4AKG) [19] is ~7.0 Å away from the W-A in the yeast motor AMPPNP (4W8F) [20]. The W-A region (Gly1974–Thr1980 in *D. discoideum* and Gly1796–Thr1803 in *S. cerevisiae*) shifts by ~1.4 Å (*Dictyostelium* motor ADP) compared to that in yeast motor AMPPNP (4W8F) [20]. The H5 (Ser1926–Ile1936) of AAA1 in the yeast motor apo (4AKG) [19] crystal structure shifts by ~3.8 Å from the position of the equivalent helix in the yeast motor AMPPNP (4W8F) [20]. The H5 helices (Arg2105–Tyr2114 in *D. discoideum* and Ser1926–Ile1936 in *S. cerevisiae*) in the *Dictyostelium* motor ADP (3VKG) [18] and in the yeast motor AMPPNP (4W8F) [20] are ~1.3 Å apart, similar to the H7 (Leu1970–Pro1982) of AAA1 in the yeast motor AMPPNP (4W8F) [20] and yeast motor apo (4AKG) [19] at a ~3.9 Å distance. There is a ~2.0 Å distance between the H7 helices (Gly2148–Lys2165 in *D. discoideum* and Leu1970–Pro1982 in *S. cerevisiae*) of the *Dictyostelium* motor ADP (3VKG) [18] and yeast motor AMPPNP (4W8F) [20]. The displacements of the domain segments (i.e., yeast motor AMPPNP [20], yeast motor apo [19], and *Dictyostelium*-motor-ADP [18]) imply that AMPPNP binding caused an “induced fit”-driven conformational change in the binding site (Figures S2 and S3).

The docked AMPPNP conformation obtained from its energy minimization (−40.18 kJ/mol) had 4.75 Å RMSD due to the optimization of the bond lengths and angles according to the implemented force-field parameters. Similar to the reference ligand, the conformation of the docked, energy-minimized (i.e., the optimized) structure of AMPPNP interacted with ATP motifs [4,7]. However, the orientation of the aromatic nucleotide fragment of the energy minimized AMPPNP allowed the system to engage with positively charged Lys1974 of the H7 (Leu1970–Pro1982) via polar ionic interactions, which is not possible for the ligand with the conformation seen in the crystal structure. Unlike the latter, the amine group of the optimized conformation engaged in H-bond interactions with the carboxylate group of Glu1767 (N-loop: Pro1766–Leu1774), and its γ-phosphate created an H-bond with Gln1849 (E1849Q) (Figure 6B,C, Table 3 and Table 5).

**Figure 7:**
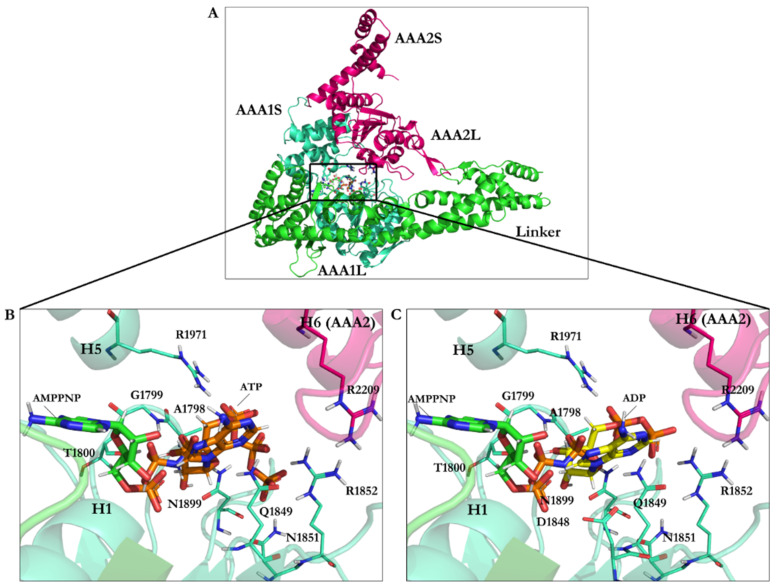
(**A**) The linker and the AAA1 and AAA2 subunits of dynein and illustration of their inter-subunit binding site. Binding interactions of docking solutions of (**B**) ATP (orange) and (**C**) ADP (yellow) compared to AMPPNP (green) at the AAA1 binding site.

The energy-minimized ATP’s binding energy is lower than that of AMPPNP, which suggests ATP binds more strongly to cytoplasmic dynein 1 than AMPPNP (−42.33 kJ/mol vs. −40.18 kJ/mol). The ATP’s binding mode obtained after the conformational search displayed its interaction with Ala1798, Gly1799, and Thr1800 from the W-A region (Gly1796–Thr1803), Gln1849 from the W-B motif in β3 (Ala1843–Asp1848), Asn1851 and Arg1852, between β3 (Ala1843–Asp1848) and H3 (Glu1854–Val1874), with the S-I (Asn1899 in β6: Ala1893–Asn1899), and Arg1971 from H7 (Leu1970–Pro1982) (Figure 7B, Table 3 and Table 5).

The ADP’s binding energy was −31.89 kJ/mol, which was the highest among the nucleotides (ATP with −42.33 kJ/mol and AMPPNP with −40.18 kJ/mol), thus presenting the lowest affinity toward the AAA1 binding site (Figure 7C and Table 5).

#### 3.2.1. Ciliobrevin A and D

The calculated conformation of ciliobrevin A (binding energy −26.23 kJ/mol) had a stronger affinity than ciliobrevin D (binding energy −23.92 kJ/mol). Ciliobrevin A (IC_50_ of 52.0 µM [26]) had a lower potency than the D analogue (IC_50_ of 15.0 µM [26]). Ciliobrevin A and D both displayed weaker binding affinity than ATP (−42.33 kJ/mol), AMPPNP (−40.18 kJ/mol), and ADP (−31.89 kJ/mol) (Figure 4 and Table 5 and Table 6).

**Figure 8:**
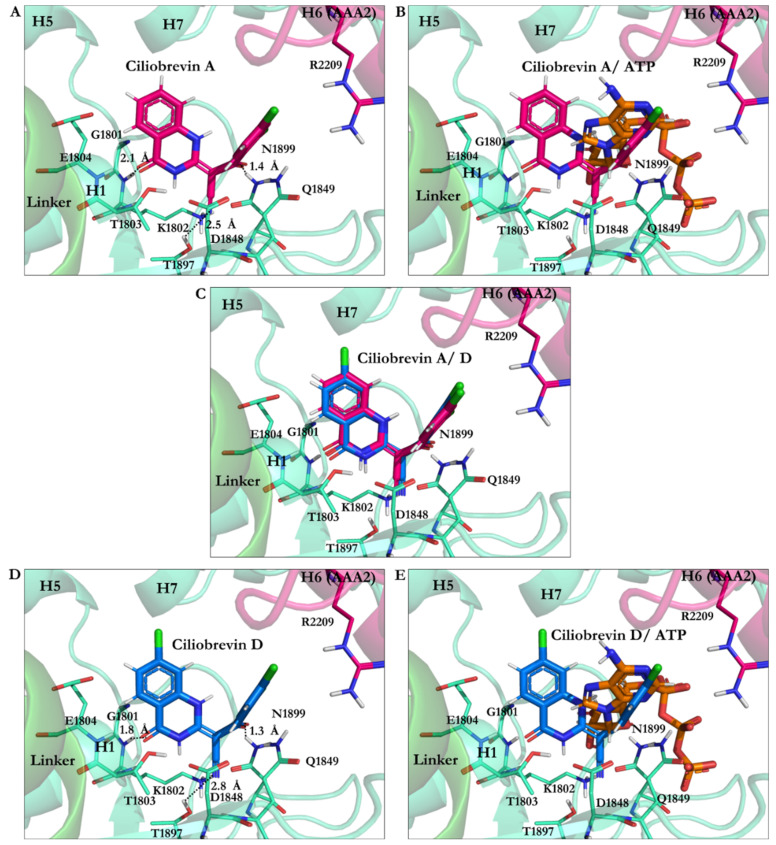
Ciliobrevin A and D conformation at the AAA1 binding site of motor domain of dynein 1. (**A**) Ciliobrevin A and (**B**) Ciliobrevin A superimposed on ATP. (**C**) Ciliobrevin A and ciliobrevin D superimposed. (**D**) Ciliobrevin D and (**E**) Ciliobrevin D superimposed on ATP.

The O21 atom of ciliobrevin A was involved in a ~2.1 Å hydrogen bond (H-bond). In contrast, the O21 of ciliobrevin D formed a ~1.8 Å H-bond with Lys1802 (the W-A motif, Gly1796–Thr1803); the positively charged ammonium fragment of Lys1802 usually contributes to the stabilization of the negatively charged ATP γ-phosphate [37]. The O22 of ciliobrevin A and D engaged in an H-bond with the side-chain of Asn1899 S-I motif of the β6 (Ala1893–Asn1899) at a ~1.3 Å–1.4 Å distance. The S-I is involved in placing a water molecule near the γ-phosphate of ATP and the negative charge of Glu1849 of the W-B motif, thereby facilitating a nucleophilic attack for hydrolyzation [37]. The O22 in ciliobrevin A and D also formed an H-bond (~1.8 Å and ~1.9 Å, respectively) with Gln1849 (in E1849Q mutant). In the wild-type dynein, Glu1849 is responsible for activating a water molecule placed by the S-I (Asn1899 in yeast dynein 1) to trigger the network mechanism of ATP hydrolysis [37]. Therefore, the E1849Q mutation in the yeast motor AMPPNP crystal structure represents a conformation incapable of ATP hydrolysis [20]. In the in silico conformational search that the mutant of dynein was studied, Gln1849 showed interactions with ciliobrevin A and D via H-bond formation. The N9 atom of the ligands interacted with the hydroxyl group of Thr1803 of the W-A motif (Gly1796–Thr1803) through a 2.8 Å H-bond in ciliobrevin A and a 2.9 Å H-bond in ciliobrevin D, while Thr1803 usually participates in the stabilization of the ATP γ-phosphate [37]. Thus, by interacting with Thr1803, ciliobrevin A and D could block the activity of the subsites, which otherwise would be involved in the hydrolytic reaction on the ATP (Figure 4 and Figure 8).

The cyanide (CN) moiety of ciliobrevin A formed an H-bond (~2.5 Å) with Thr1897 of β6 (Ala1893–Asn1899). The CN was involved with the hydroxyl (OH) moiety of Thr1897 in ciliobrevin D (at ~2.8 Å distance). It is noteworthy that Thr1897 does not belong to the ATP motifs, nor did it show any interactions in the in silico docking solutions of the nucleotides. However, the CN moiety seemed to act as an auxiliary anchor to promote placements and orientations of the significant substructures of ciliobrevin A and D in the proximity of the critical ATP motifs, namely, the W-A and the S-I. Aliphatic chains of the W-A (by Gly1799, Lys1802), the S-I (by Asn1899), and the W-B motifs (by Asp1848 and Gln1849) were involved in van der Waals (VdW) interactions with the hydrophobic fragments of ciliobrevin A and ciliobrevin D. This suggests how ciliobrevin A and D’s effects as ATP antagonists, on the ATP motifs and Thr1897, could disturb the activity of the motor domain by blocking the catalytic residues’ action (Figure 8 and Table 3).

#### 3.2.2. The Analogues Binding Profile

**Figure 9:**
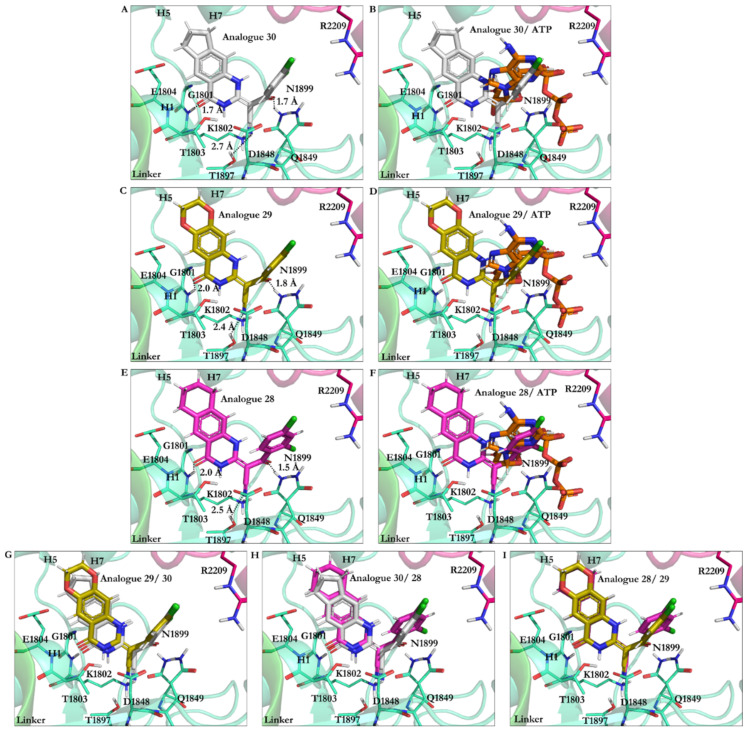
Analogues of ciliobrevin at the AAA1 binding site of dynein 1. (**A**) Analogue 30. (**B**) Analogue 30 superimposed on ATP. (**C**) Analogue 29. (**D**) Analogue 29 superimposed on ATP. (**E**) Analogue 28. (**F**) Analogue 28 superimposed on ATP. Superimposition of (**G**) analogues 29 and 30, (**H**) analogues 30 and 28, and (**I**) analogues 28 and 29.

Analogue 30 showed the best affinity, along with analogues 29 and 28 (the lowest energy −27.87 kJ/mol vs. respective −27.37 kJ/mol and −27.27 kJ/mol). The O21 in analogues 28, 29, and 30 was involved in an H-bond with the polar H of the amide bond moiety of Gly1801 in the ATP motif, the W-A (Gly1796–Thr1803). Furthermore, their N9 atom formed an H-bond with the OH moiety of Thr1803 of the W-A (Gly1796–Thr1803). In these analogues, the CN moiety played a similar role in ciliobrevin A and D. It was also involved in an H-bond formation with the OH of Thr1897 in the β6. The side-chains of Asn1899 in the S-I and Gln1849 of the W-B motif also created an H-bond with the O22 of the analogues. The hydrocarbon chains of Gly1799 and Lys 1802 in the W-A motif, as well as Asp1848 and Gln1849 of the W-B motif, hydrophobically interacted with the C8 of quinazolinone ring B and the acrylonitrile moiety. Thus, analogues 28–30 engaged with ATP motifs and the β6, through which they could hinder the motor domain’s natural function (Figure 5 and Figure 9, Table S1).

Chemical modifications resulting in analogue 45 showed its improved binding affinity versus analogues 28–30, 38, and 42, as well as ciliobrevin A and D. However, the similarity in their binding profiles showed that they could comparably compete with ATP for binding to the functional motifs in the AAA1 nucleotide-binding site. The analogues’ O21 atom formed an H-bond with the polar H of the Gly1801 in the W-A motif, and their N9 atom formed an H-bond with the OH of Thr1803 in the same ATP motif. There was also an H-bond between the O22 and Asn1899 of the S-I and Gln1849 of the W-B. Similar to analogues 28–30, the CN moiety of analogue 42 formed an H-bond with Thr1897 of the β6. Thr1897, which does not belong to the ATP motifs, also interacted with ciliobrevin A and D, as well as analogues 45, 30, 42, 29, 28, and 38. The OH of Thr1897 was involved with Gln1849 (in the W-B motif), known for its connection with water molecules to promote ATP hydrolysis. In analogue 38, the CN was replaced with a methoxy (–OMe) moiety. The O34 of the methoxy group interacted with Lys1974 via a 2.2 Å H-bond. The benzene ring was replaced with pyridine in analogue 45, whose N31 atom formed a 2.1 Å H-bond with Lys1974 in the H7 helix (Leu1970–Pro1982). The Lys1974 positive charge could potentially form a dipole-induced moment with the pyridine ring of analogue 45, although the positively charged amino acid is not perpendicular to the ring (Table S2, Table 3 and Figure S6).

The analogues of ciliobrevin (i.e., ciliobrevin A and D, as well as analogues 28, 29, 30, 38, 42, and 45) affect the ATP hydrolysis process also through binding to Asn1899 (in the S-I), which usually forms an H-bond with and positions water molecules for the nucleophilic substitution [37]. A conserved Asn residue (e.g., Asn64 in PspF, a member of the AAA+ proteins [38]) is involved in an H-bond formation with the conserved Glu from the W-B motif (Glu108 in PspF of AAA+ proteins [38]) found at the AAA1 binding site of several dyneins [37]. Through the interactions of an ATP competitive inhibitor with the Glu or the Asn, the Asn (Asn64 in PspF [38]) cannot contribute to the ATP hydrolysis, as the glutamate residue of the W-B motif (Glu108 in PspF [38]) is unavailable to activate a water molecule through deprotonation [37]. The process is referred to as a “glutamate switch” and is thought to be an endogenous mechanism that regulates ATP hydrolysis in dynein to evade a nonproductive powerstroke [37]. H-bond formations of Asn1899 with ciliobrevin A and D, as well as its analogues, could disrupt the “switch” mechanism and, therefore, interfere with the regulation of the dynein powerstroke progression. The glutamate switch involving Glu108 in PspF [38] has not yet been detected in cytoplasmic dynein 1. The Asn residue involved in the “glutamate switch” is replaced with a cysteine (Cys1822 in *S. cerevisiae*) in the dynein 1 isoform [37]. However, an intramolecular H-bond of 2.1 Å between the side-chains of Gln1849 (E1849Q) and Arg1852 was visualized in the optimized (i.e., energy-minimized) structure of yeast motor AMPPNP dynein obtained through an in silico conformational search. In contrast, the crystal structure shows a relatively long distance (3.9 Å) between the residues. Thus, the energetically stabilized conformation demonstrated Arg1852 and Gln1849 in the positions and orientations capable of strong H-bond formation, where an arginine in place of the asparagine could interact with the glutamate to execute the “switch” mechanism in dynein 1 (Figure S7).

#### 3.2.3. Geometrical Isomerization Effect on Ciliobrevin Binding to the AAA1

Ciliobrevin A and D exist in two geometric isomers of *E* or *Z* at the C8–C11 double bond [10]. The potency of the ciliobrevin was thought to be affected by isomerization, where there is only a fraction of the isomer abrogating dynein [4]. The benzoylacrylonitrile group of the molecule favors the *E* isomer since the one-dimensional NMR spectrum and the result of a 2D nuclear Overhauser effect spectroscopy (NOESY) of ciliobrevin D showed an intramolecular H-bond between the hydrogen atom on the N7 and the O22 that stabilizes ciliobrevin D in solution [26]. The N7 is directly attached to the C8, and the O22 relates to the C11 via the double bond to the C13, which is covalently attached to the C11 (Figure 5 and Figure S8).

**Figure 10:**
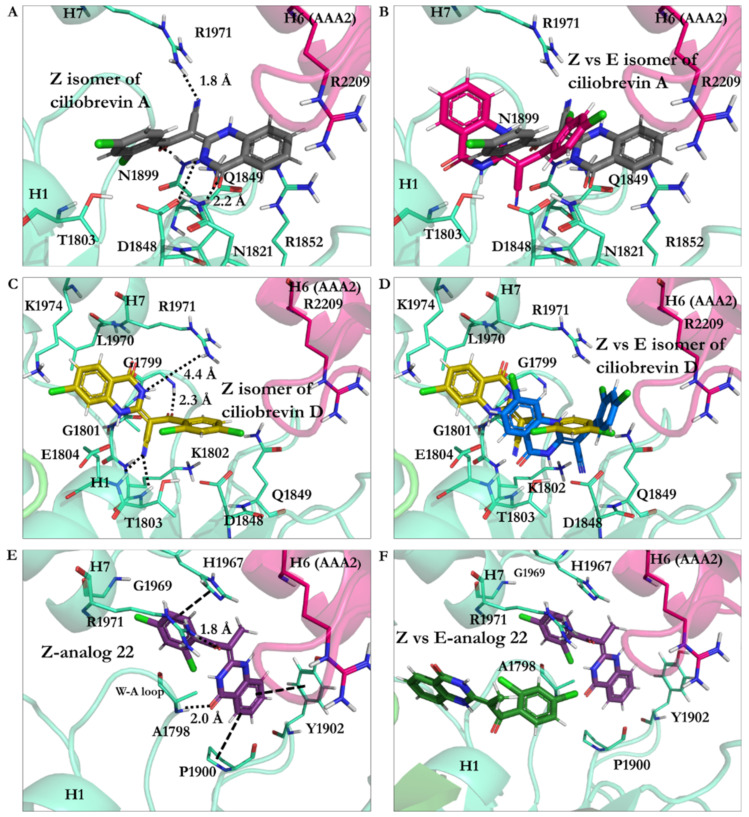
(**A**) *Z* isomer of ciliobrevin A at the AAA1 binding site of cytoplasmic dynein 1. (**B**) Superimposition of *Z* and *E* isomers of ciliobrevin A. (**C**) *E* isomer of ciliobrevin D at the AAA1 binding site of cytoplasmic dynein 1. (**D**) *E* isomer of ciliobrevin D superimposed on its *Z* isomer. (**E**) *Z* analogue 22 and the residues at the AAA1 binding site of AAA1. (**F**) *Z* analogue 22 superimposed on its *E* isomer at the AAA1 binding site.

*Ciliobrevin A*: The effect of geometrical isomerization of ciliobrevin A was investigated, where its *Z* isomer showed binding with 6.82 kJ/mol higher energy than the *E* isomer (−26.23 kJ/mol). The O22 atom of the *Z* isomer engaged in a 1.9 Å H-bond with the side-chain of the S-I, through Asn1899 in β6 (Ala1893–Asn1899), and a 2.7 Å H-bond with β3 (Ala1843–Asp1848) of the W-B via Gln1849. The *Z* isomer did not interact with Thr1897 of the β6 (Ala1893–Asn1899), unlike the *E* isomer of ciliobrevin A. On the other hand, its CN moiety formed an H-bond with the S-II ATP motif through Arg1971 of the H7 helix (Leu1970–Pro1982). The N9 atom of the *Z* isomer was involved in a 2.2 Å H-bond with the carboxylate moiety of Asp1848 from β3 (Ala1843–Asp1848), and its O21 atom also formed a 2.2 Å H-bond with the amino group of Asn1821 in the β2 strand (Val1818–Asn1821). Ring A of the *Z* isomer oriented to form a π–π stacking with the guanidine moiety of Arg1852, a key element in the “glutamate switch” in dynein 1, as observed in the conformation obtained in this in silico conformational search. The binding energies of the *E* and *Z* isomers of ciliobrevin A indicated that the *E* is favorable over the *Z* isomer, as the former displayed a significantly higher binding affinity toward the AAA1 binding site (Figure 10A,B and Table S3).

*Ciliobrevin D*: The binding energy of its *Z* isomer, similar to ciliobrevin A, was also higher than its *E* (−19.78 kJ/mol vs. −23.92 kJ/mol, respectively). The *Z* isomer utilized its O22 atom to engage in an H-bond with the polar hydrogen of the Gly1799 amide moiety in the W-A motif (Gly1796–Thr1803). The *Z* isomer’s CN group was available to form H-bonds with Lys1802 (1.9 Å) of the W-A motif, as well as Thr1803 (2.5 Å) and Glu1804 (2.3 Å). The N9 of the *Z* isomer formed a weak H-bond with Arg 1971 in the H7 helix (Leu1970–Pro1982). It is noteworthy that the *Z* isomer of ciliobrevin D did not interact with the S-I motif via Asn1899 (Figure 10C,D and Table S3).

The effect of the CN elimination from ciliobrevin A and its replacement with a methyl group in analogue 22 [7] was examined through the study of its geometric isomers. The *E* isomer became weaker than *E*-ciliobrevin A and D; however, it was slightly stronger than its *Z* isomer (analogue 22 *E* isomer, −17.49 kJ/mol, versus −16.83 kJ/mol). The *E* was bound to the AAA1 site via H-bond with Lys1802. It also formed an H-bond via its N9 atom with the carboxylate group of Glu1804. The *Z* isomer of analogue 22 displayed a ~180° rotation in the binding site compared to its *E* isomer and ciliobrevin A and D. Its unique orientation resulted in H-bonds with Ala1798 and Arg1971 via its O22 atom. In addition, His1967 interacted with its ring D of the *Z* isomer through a T-shaped π–π stacking. Its ring A also showed a similar conformation against Tyr1902, while the Pro1900 orientation facilitated a proline–benzene VdW interaction via the ligand’s ring D (Figure 10F and Table S3).

#### 3.2.4. Dynapyrazole A and B

**Figure 11:**
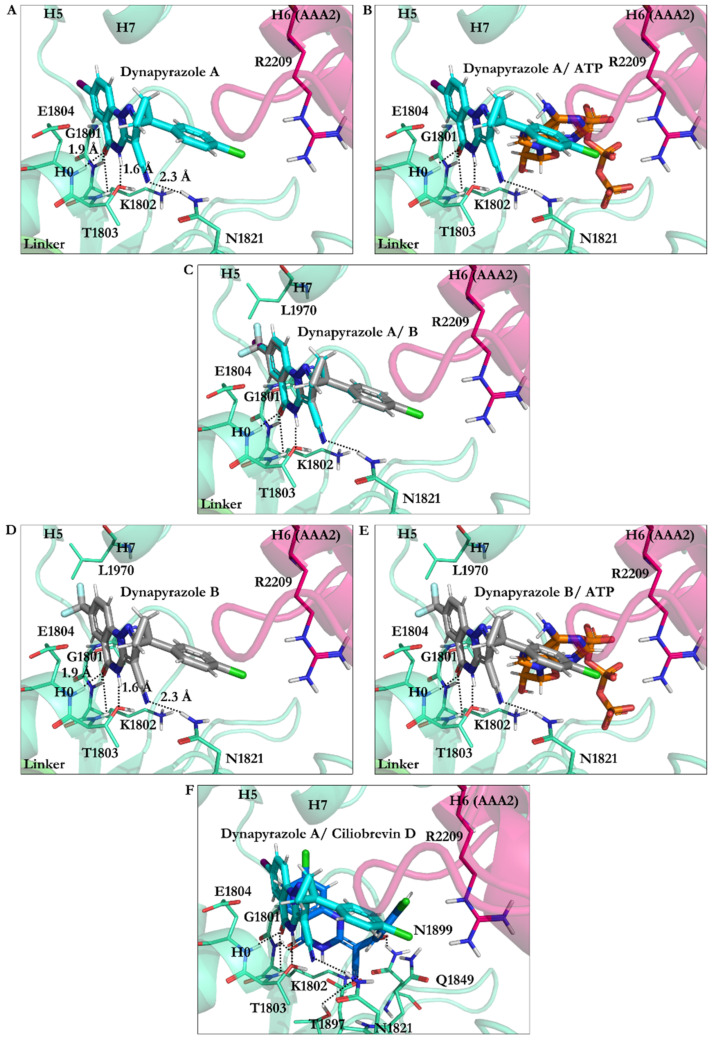
Dynapyrazole in the nucleotide-binding site of the AAA1 (**A**) dynapyrazole A. (**B**) Superimposition of dynapyrazole A and ATP. (**C**) Superimposition of dynapyrazole A and B. (**D**) Binding modes of dynapyrazole B. (**E**) Superimposition of dynapyrazole B and ATP. (**F**) Superimposition of dynapyrazole A and ciliobrevin D.

The O21 of dynapyrazole A and B formed H-bonds (~1.9 Å) with the W-A peptide backbones (Gly1796–Thr1803) via Lys1802 and the H1 helix (H1, Glu1804–Gly1810). The H atom on the N9 in dynapyrazole A and B was involved in a 1.6 Å H-bond with the W-A via Thr1803, whose OH moiety typically interacts with an Mg^2+^ resulting in stabilizing the charges on the ATP γ-phosphate [37]. Thus, the ligands, which have a slight binding difference (~0.32 kJ/mol), could similarly hinder the interaction between the cation and the Thr. In addition, the CN moiety of dynapyrazole A and B formed H-bonds with the β2 strand (Val1818–Asn1821) through Asn1821 (2.3 Å), which was ~4.0 Å away from Asp1848, a member of the W-B motif in the β3 (Ala1843–Asp1848). They could indirectly affect dynein’s motility affecting the W-B’s Asp1848, a segment that usually hosts ATP to undergo hydrolysis [4,37] (Figure 11 and Table S4).

#### 3.2.5. Impact of Elimination of Carbon Double Bond on the Affinity of Dynapyrazole and Analogues

Ciliobrevin’s derivatization led to the synthesis of dynapyrazole by eliminating the C8–C11 double bond and inserting the ring C in dynapyrazole and its analogues [26]. The process also consisted of replacing the O22 atom in ciliobrevin with the N11 in dynapyrazole to improve its potency. That resulted in the IC_50_ plummeting from 15 µM (ciliobrevin D) to 2.3 µM (dynapyrazole A) [26], whereas the binding strength of the former improved by ~−5 kJ/mol (Figure 4 and Table 4, Tables S3 and S4).

The double bond in ciliobrevin A and D positionally allowed the *E* isomer to form H-bonds both with Thr1897 of the β6 (Ala1893–Asn1899) via the nitrogen of its CN moiety and with Asn1899 through its O22 atom. The energy contribution of this event could have been the cause of the difference in the total binding strength, considering that the N11 of the replaced ring C in dynapyrazole had no interaction with the AAA1 binding site, in contrast to the eliminated O22 in ciliobrevin. However, the CN nitrogen atom of dynapyrazole A and B formed an H-bond with Asn1821 (Figure 11F).

**Figure 12:**
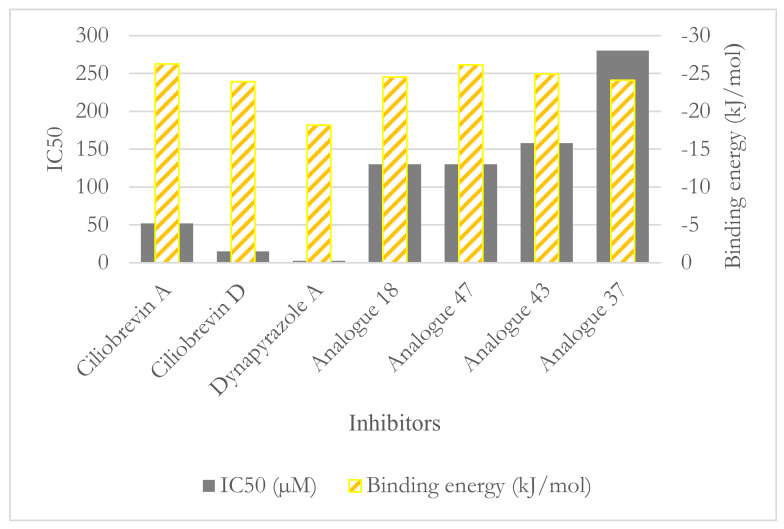
The ligands binding energy versus their IC_50_.

Among the analogues 37, 43, and 47 of ciliobrevin possessing the double bond, analogue 47 showed the lowest IC_50_ (130.0 µM [26]) and the strongest binding (−26.12 kJ/mol), whereas analogue 37 with the highest IC_50_ (280.0 µM [26]) had just 2.01 kJ/mol higher binding energy than analogue 47. Analogue 47 is suggested as the most suitable candidate for further in vitro and in vivo experimental evaluations for its effect on dynein motility and its selectivity profile (Figure 12).

#### 3.2.6. Protonation Effect on Ciliobrevin A and D Binding

Considering the pKa values of the chemical moieties of the inhibitors, as stated earlier, the N9 and N7 might be weak candidates for protonation at the tissues with alkaline pH (Figure 4, Figure S9, and Table 6).

*Protonation of ciliobrevin A at the N9 position* caused a ~−2.0 kJ/mol improvement in binding strength, suggesting that ciliobrevin A might be protonated at the N9 depending on the environmental pH, which would interfere with the motor function. However, the possibility seems low concerning the juxtaposed carbonyl moiety at the C10. Unlike the neutral (unprotonated) ciliobrevin A, its protonated form interacted with Ala1798 of the W-A motif (Gly1796–Thr1803) through its benzylic ring D. The protonated N9 atom was 2.6 Å away from the carboxylate moiety of Asp1848 in the β3 (Ala1843–Asp1848), which could have also electrostatically affected the positively charged N9 atom and contribute to the strengthening of ciliobrevin A binding affinity.

*The protonated N9 of ciliobrevin D* projected a similar binding profile to that of the A analogue with enhanced binding compared to its neutral form (−23.92 kJ/mol vs. −26.15 kJ/mol). Therefore, ciliobrevin D, protonated under a proper pH, had a superior inhibitory effect on dynein 1 in vitro. 

*The protonated ciliobrevin A at the N7* was a weaker binder (~2.39 kJ/mol) compared to its neutral structure (with −26.23 kJ/mol), suggesting that the ligand is less likely to be protonated at the N7 position in solution, as the nitrogen atom’s lone-pair electrons tend to participate in the delocalized electron cloud of the aromatic ring A.

*Protonation of the N7 atom of ciliobrevin D* had a minor effect on binding (0.37 kJ/mol), since protonated and unprotonated D analogues similarly treated the AAA1 nucleotide site through Gly1801, Lys1802, Thr1803, Glu1804, Asp1848, Gln1849, Thr1897, and Asn1899.

#### 3.2.7. Effect of Protonation on Binding Mode of Dynapyrazole A and B

*Protonation of dynapyrazole A at N9* resulted in a slight binding improvement (~−0.64 kJ/mol). This analogue was the only one in the library of 63 ligands to bind to the linker domain of dynein. An ionic interaction was formed between the protonated N9 and the carboxylate group of Glu1586 in the T-turn 6 (Val1586–Glu1588 of the linker), while its O21 formed an H-bond with the amide moiety of Pro1766 in the N-loop (Pro1766–Leu1774). The aliphatic side-chains of Lys1696 and Glu1699 in the H13 of the linker domain (Asp1692–Asn1717) and Glu1767 in the N-loop (Pro1766–Leu1774) were hydrophobically affected by the hydrocarbon fragment of the ligand consisting of the C8, the C12, and the C13 atoms. The positively charged guanidine moiety of Arg1978 in the H7 (Leu1970–Pro1982) interacted with the monochloride benzylic ring D through polar interactions. These observations elucidated the improvement of the total binding strength of the N9-protonated dynapyrazole A.

**Figure 13:**
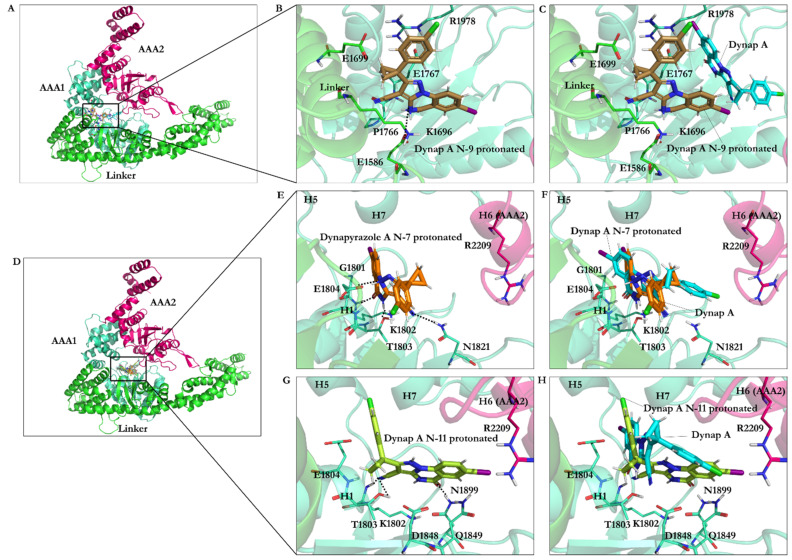
Binding of the protonated dynapyrazole A at the AAA1 binding site of dynein 1. (**A**) Overview of the AAA1 and AAA2 units, and linker domains. (**B**) Dynapyrazole A protonated at the N9 atom interacting with the linker residues. (**C**) Dynapyrazole A protonated at the N9 atom superimposed on dynapyrazole A. (**D**) Overview of the AAA1 and AAA2 units, and linker subdomains (as the panel A). (**E**) Dynapyrazole A protonated at the N7 atom. (**F**) Dynapyrazole A protonated at the N7 atom superimposed on dynapyrazole A. (**G**) Dynapyrazole A protonated at the N11 atom. (**H**) Dynapyrazole A protonated at the N11 atom superimposed on dynapyrazole A.

*Protonation of dynapyrazole A at N7* also benefited from protonation (~−8.72 kJ/mol). The considerable improvement indicated that the IC_50_ in vitro better correlated with the ligand’s protonated at the N7 position. It electrostatically interacted with the negative charge of Glu1804 carboxylate in the H1 helix (Glu1804–Gly1810), while its N9 created an H-bond to the OH moiety of Thr1803 in the W-A (Gly1796–Thr1803). The data showed that protonation at the N7 site was beneficial to the ligand (Figure 4 and Figure 13 and Table S4).

*Protonation of N11 in dynapyrazole A* caused slight weakness of the binding (0.27 kJ/mol) compared to its neutral form due to a minor difference of the interaction network set up by the N11-protonated ligand. The protonated N11 atom showed no ionic interactions. Dynapyrazole A in this configuration was the only analogue, among the protonated and neutral dynapyrazole A and B, to interact with the β6 (via Asn1899) and the β3 strand (via Asp1848 and Gln1849), resembling the binding mode of ciliobrevin and its analogues.

*Protonation of dynapyrazole B at N11* also had an insignificant effect on its binding (~0.62 kJ/mol), similar to its protonated A analogue. It had the lowest predicted affinity toward the AAA1 subunit in the ligands library and involved Val1819 through VdW forces via its ring D. In summary, protonation at the N11 was disadvantageous to dynapyrazole A and B and weakened their binding affinities to the AAA1 site (Figure 4, Figure S10E,F, and Table S4).

## 4. Conclusions

The presented work provides structural data according to an SAR study to explain how ciliobrevin A and D, dynapyrazole A and B, and their protonated structures, as well as the 46 analogues, could inhibit ATP binding and its hydrolysis in the nucleotide-binding site of the AAA1 subunit of the motor domain in cytoplasmic dynein 1. The lowest binding energy of ATP among the 63 ligands of the library suggested its superior binding affinity over all the competitive inhibitors. However, ciliobrevin A and D, as well as most of the analogues could bind to the functionally key subsites, including the Sensor I and II, N-loop, and the W-A and B, also known as the ATP motifs; thus, optimizing the concentration of the competitive inhibitors in vitro could result in blocking the AAA1 nucleotide site in the absence of ATP or its lower concentration. In particular, analogue 47 is suggested as the most suitable candidate for further in vitro and in vivo experimental evaluations due to its strong binding affinity and low IC_50_. The ligands’ structural mechanism of interference with the ATP binding and hydrolysis was shown to vary depending on their critical functional fragments. The presence of the carbonyl oxygen on ring B of the ligands, for instance, in ciliobrevin D, resulted in its O21 atom forming an H-bond with the Lys1802 amine moiety in the W-A motif. The positively charged ammonium group of the Lys usually acts as an anchor by applying electrostatic forces on the negatively charged γ-phosphate, thereby contributing to the catalytic network for ATP hydrolysis. At the same time, the O22 of the ligand formed an H-bond with Asn1899 of the S-I motif in the β6 strand. The S-I is involved in placing a water molecule near the γ-phosphate of ATP and the negative charge of Glu1849 of the W-B motif and enables a water molecule for a nucleophilic attack, required for the ATP hydrolyzation. The O22 also facilitated the ligand’s hydrogen bond formation with the Gln1849 in the Glu1849Gln protein mutant.

Eliminating the C8–C11 double from ciliobrevin, removing O22 and, replacing it with the N11 by insertion of the ring C in dynapyrazole, resulted in the alteration of the chemical structure, which lowered the IC_50_ in dynapyrazole. However, the N11 of the ring did not mimic the O22 effect and diminished dynapyrazole binding strength, despite being at a relatively similar position in the ligand structure. Protonation at the N11 atom did not enhance its contribution to the binding energy, as shown in a separate attempt. However, dynapyrazole A benefited from the N7 and N9 atoms’ protonation according to the improvement gained in their binding energy. The N9-protonated dynapyrazole A was the only analogue in the ligand library to bind to the linker domain of dynein. The ligand conformational pose and the consequent binding to the linker domain were facilitated by the electrostatic interaction between the protonated N9 and the carboxylate group of Glu1586 in the T-turn 6 of the linker and an H-bond with the amide moiety of Pro1766 in the N-loop. The aliphatic side-chains of the H13 helix in the linker domain, as well as the N-loop, interacted with the ligand hydrophobic sites, namely, the C8, the C12, and the C13 atoms. The observation explained the improvement of the total binding strength of the N9-protonated dynapyrazole A against its unprotonated form.

There are two geometrical isomers *E* and *Z* of ciliobrevin, according to its C8–C11 double bond. The *E* isomer enabled H-bond formation of the ligand with the β6 via its Thr1897 through the nitrogen of the ligand CN moiety and with Asn1899 via its O22 atom. The *Z* isomer of the analogue D interacted with the W-A motif, showing no substantial effect on the S-I motif. In contrast, the *Z* isomer of ciliobrevin A interacted with the S-I motif through Asn1899 of the β6 and the β3 strand, as well as with the W-B via Gln1849. Unlike its *E* isomer, the *Z* of ciliobrevin A showed no effect on Thr1897 of the β6. However, its CN moiety caused an H-bond with the S-II motif. Ring A, the benzene moiety of the *Z* isomer, had a polar interaction with Arg1852 in a position suitable for a π–π stacking with the guanidine moiety of the arginine. It also appeared to contribute to the “glutamate switch” mechanism in dynein 1. The binding energies of the geometric isomers of ciliobrevin A and D indicated that the *E* had a significantly higher affinity than the *Z* toward the AAA1 binding site. Assessing the effect of geometrical isomerization on analogue 22 resulted in the conformation of its isomers in two opposite orientations in the binding site. This was likely due to the replacement of the CN moiety with a methyl group in that particular analogue, causing a drastic change in the isomer binding modes. 

The benzene ring replaced with a pyridine moiety in analogue 45 led to a polar interaction with Lys1974. Analogue 45, similar to other pyridine-possessing analogues of ciliobrevin (i.e., 28, 29, 30, 38, 42, and 45) could affect the ATP hydrolysis via binding to Asn1899, a conserved residue of the S-I motif that facilitates placing water molecules for nucleophilic substitution in ATP hydrolysis.

The glutamate switch (involving Glu108 in PspF [38]) has not yet been detected in cytoplasmic dynein 1; however, an intramolecular H-bond of 2.1 Å between the side-chains of Gln1849 (E1849Q) and Arg1852 was detected in the most energetically favorable conformation of the yeast motor domain through the in silico conformational search. It exhibited a conformation with Arg1852 and Gln1849 in orientations capable of a close and strong H-bond formation, suggesting that arginine could also interact with the glutamate in regulating the “switch” mechanism in dynein 1.

New analogues of dynapyrazole, recently introduced by Santarossa et al., [36] were shown to be potent in inhibiting basal ATPase activity of dynein while binding to AAA3 and AAA4. The binding assessment of analogues 3–48 will be critical in evaluating their inhibitory mode of action at the AAA3 binding site, which is not conserved in axonemal dynein and cytoplasmic dynein 2. This makes the AAA3 subunit a suitable target in the future direction of this study. Utilizing the presented information can contribute to setting up experiments with a focus on the most promising analogues for their selectivity to dynein 1 versus its second isoform. This research suggests avenues to improve the potency and selectivity of the small-molecule inhibitors that could target cytoplasmic dynein’s activity to treat neurodegenerative and cancer diseases.

## Figures and Tables

**Table 1:** Summary of the features of the crystallographically solved structures of dynein used in this study.

PDB Code	Uniprot Code	Species	Resolution (Å)	Exp. pH	Nucleotide Binding Domain	Missing Residues	Released Date
**4AKG**	P36022	*S. cerevisiae*	3.3	5.6	AAA1 (apo), AAA2 (ATP), AAA3 (ADP)	2944–2959 (AAA4) and 3658–3669 (AAA5–AAA6)	14 March 2012
**4W8F**	P36022	*S. cerevisiae*	3.54	8.0	AMPPNP in AAA1, AAA2, AAA3 and AAA4	2025–2029 (AAA1–AAA2), 2950–2953 (AAA4), 3659–3668 (AAA5–AAA6)	12 December 2014
**3VKG**	P34036	*Dictyostelium discoideum*	2.81	7.0	ADP in AAA1, AAA2, AAA3 and AAA4	2061–2063 (AAA1), 2454–2488 (AAA2), 3212–3215 (AAA4), 3699–3703 (AAA5), 3725–3758 (AAA5), 4114–4115 (AAA6)	14 March 2012

**Table 2:** Amino-acid sequence identity and similarity of the different parts of dynein, between *S. cerevisiae* and *D. discoideum*.

Dynein (*S. cerevisiae* and *D. discoideum*)	Sequence Identity (%) (No. of Residues)	Sequence Similarity (Residues)
**Entire amino-acid sequence of cytoplasmic dynein**	24.83% (1193 residues)	1668
**AAA1**	52.02% (116 residues)	63
**AAA2**	28.14% (83 residues)	95
**AAA1 and AAA2**	34.67% (207 residues)	185

**Table 3:** The ATP motifs in *S. cerevisiae* and *D. discoideum*.

ATP Motifs	*S. Cerevisiae*	*D. Discoideum*
**Walker-A**	Gly1796–Thr1803	Gly1974–Thr1980
**Walker-B**	Asp1848–Glu1849	Asp2026–Glu2027
**Sensor I**	Asn1899	Asn2078
**Sensor II**	Arg1971	Arg2150
**Arg finger**	Arg2209	Arg2410
**N-loop**	Leu1769–Ile1770	Leu1947–Val1948

**Table 4:** IC_50_ values of ciliobrevin A and D, their analogues 18, 37, 43, and 47, and dynapyrazole A and B for dynein 1 and dynein 2.

Compounds	IC_50_ (µM) Dynein 1	IC_50_ (µM) Dynein 2
**Ciliobrevin A**	52.0	55.0
**Ciliobrevin D**	15.0	15.5
**Dynapyrazole A**	2.3	2.6
Dynapyrazole B *	_	2.9
**Analogue 18**	130.0	21.0
**Analogue 37**	280.0	11.0
**Analogue 43**	158.0	16.0
**Analogue 47**	130.0	11.0

* The IC_50_ of dynapyrazole B against dynein 1 is not available.

**Table 5:** Binding properties of the nucleotides’ conformation obtained from the in silico experiments.

Ligand	RMSD vs. X-ray Structure	Binding Energy (kJ/mol)	Residues Interacting with the Compound
**AMPPNP**	1.67	−22.06	Leu1769, Ile1770, Gly1799, Gly1801, Lys1802, Thr1803, Glu1804, Asn1899, Ile1929, Leu1970, Lys1974
**AMPPNP** **energy-minimized structure**	4.75	−40.18	Glu1767, Gly1799, Gly1801, Lys1802, Thr1803, Glu1804, Gln1849, Asn1899, Lys1974
**Minimized ATP**	—	−42.33	Ala1798, Gly1799, Thr1800, Gln1849, Asn1851, Arg1852, Asn1899, Arg1971
**Minimized ADP**	—	−31.89	Ala1798, Gly1799, Thr1800, Asp1848, Gln1849, Arg1852, Asn1899, Arg1971

**Table 6:** Amino acids affected by ciliobrevin A and D in their protonated and deprotonated states.

Compound	Binding Energy (kJ/mol)	Residues Interacting with the Compound
**Ciliobrevin A N9 protonated**	−28.22	Ala1798, Lys1802, Thr1803, Glu1804, Asp1848, Gln1849, Thr1897, Asn1899, Arg1971
**Ciliobrevin A**	−26.23	Gly1801, Lys1802, Thr1803, Glu1804, Asp1848, Gln1849, Thr1897, Asn1899
**Ciliobrevin D N9 protonated**	−26.15	Lys1802, Thr1803, Glu1804, Asp1848, Gln1849, Thr1897, Asn1899
**Ciliobrevin D**	−23.92	Gly1801, Lys1802, Thr1803, Glu1804, Asp1848, Gln1849, Thr1897, Asn1899
**Ciliobrevin A N7 protonated**	−23.84	Gly1799, Thr1800, Gly1801, Lys1802, Thr1803, Glu1804, Leu1970, Arg1971
**Ciliobrevin D N7 protonated**	−23.55	Gly1801, Lys1802, Thr1803, Glu1804, Asp1848, Gln1849, Thr1897, Asn1899

## Data Availability

The atomic coordinates will be available upon request.

## Supplementary Materials

The following are available online at https://www.mdpi.com/article/10.3390/ijms22147704/s1, Figure S1. Amino-acid sequence alignment of the AAA1 and AAA2 subunits of dynein corresponding to *S. cerevisiae* (1758–2273) and *D. discoideum* (1936–2531) with the ClustalW program. The W-A motif is highlighted in blue. The gray highlights represent sequence similarity. A singular dot (.) indicates that residues belong to a different group of amino acids, while a colon (:) indicates that residues belong to the same group of amino acids, and the asterisk (*) indicates sequence identity; Figure S2. (A) Structural alignment of the AAA1 and AAA2 of yeast AMPPNP (4W8F) and yeast apo (4AKG). (B) A close-up of the AAA1 binding site. Helices (H0, H1, H5, and H7) of the AAA1 subdomain, along with the Walker-A (W-A) loops, with the AMPPNP in the ball-and-stick representation in green located at the AAA1 nucleotide-binding site and the H6 of the AAA2 subdomains of the yeast AMPPNP (4W8F) and yeast apo (4AKG). Color code: yeast AMPPNP (AAA1 in green cyan and AAA2 in sharp pink) and yeast apo (AAA1 in orange and AAA2 in yellow); Figure S3. (A) Structural alignment of the AAA1 and AAA2 subunit of the *Dictyostelium* ADP (3VKG) and yeast AMPPNP (4W8F). (B) A close-up of the AAA1 binding site. Helices (H0, H1, H5, and H7) of the AAA1 subdomain, along with the Walker-A (W-A) loops, with the AMPPNP in ball-and-stick representation in green and ADP in ball-and-stick representation in yellow, located at the AAA1 nucleotide-binding site and the H4 of the AAA2 subdomain of the *Dictyostelium* ADP (3VKG) and the H6 of the AAA2 subdomain of the yeast apo (4AKG). (C) Structural alignment of ligands at the AAA1 nucleotide-binding site of the *Dictyostelium* ADP (3VKG) and yeast AMPPNP (4W8F), along with AMPPNP in ball-and-stick representation in green and ADP in ball-and-stick representation in yellow with a cross (x) on its atoms to distinguish them from the atoms of AMPPNP. Color code: *Dictyostelium* ADP (AAA1 in green and AAA2 in blue) and yeast AMPPNP (AAA1 in green cyan and AAA2 in sharp pink); Figure S4. (A) AMPPNP interactions and residues of the AAA1 nucleotide-binding site. Distances between catalytic residues of the AAA1 nucleotide-binding site of dynein and the β-phosphate of (B) AMPPNP and (C) ADP; Figure S5. Annotation of the secondary structure of AAA1 and AAA2 domains of cytoplasmic dynein according to the 4W8F crystal structure retrieved from the PDB; Figure S6. Binding modes of ciliobrevin analogues at the AAA1 binding site of dynein 1. (A) Analogue 45. (B) Analogue 45 superimposed on ATP. (C) Analogue 42. (D) Analogue 42 superimposed on ATP. (E) Analogue 38. (F) Analogue 38 superimposed on ATP. Superimposition of (G) analogues 42 and 45, (H) analogues 38 and 45, and (I) analogues 38 and 42; Figure S7. Intramolecular interactions between the catalytic residues at the AAA1 binding site and Arg1852 involving a potential glutamate switch. The docking solution of AMPPNP is represented in green sticks. Thr1803 and Asp1848 accommodate Mg^2+^ during ATP hydrolysis. Sensor I (N1899), W-B (E1849Q), and R-finger (R2209) play catalytic roles during ATP hydrolysis; Figure S8. *E* and *Z* configurations of ciliobrevin D. Red arrows point to the double bond between the C8 and C11 atoms; Figure S9. Binding modes of the protonated forms of ciliobrevin at the AAA1 binding site of dynein 1. (A) Ciliobrevin A protonated at the N9 atom. (B) Ciliobrevin A protonated at the N9 atom superimposed on ciliobrevin A. (C) Ciliobrevin A protonated at the N7 atom. (D) Ciliobrevin A protonated at the N7 atom superimposed on ciliobrevin A. (E) Ciliobrevin D protonated at the N9 atom. (F) Ciliobrevin D protonated at the N9 atom superimposed on ciliobrevin D. (G) Ciliobrevin D protonated at the N7 atom. (H) Ciliobrevin D protonated at the N7 atom superimposed on ciliobrevin D; Figure S10. Main binding interactions of the protonated forms of dynapyrazole B at the AAA1 binding site of dynein 1. (A) Dynapyrazole B protonated at the N7 atom. (B) Dynapyrazole A protonated at the N7 atom superimposed on dynapyrazole B. (C) Dynapyrazole B protonated at the N9 atom. (D) Dynapyrazole B protonated at the N9 atom superimposed on dynapyrazole A. (E) Dynapyrazole B protonated at the N11 atom. (F) Dynapyrazole B protonated at the N11 atom superimposed on dynapyrazole B; Table S1. Networking interactions of analogues 28, 29, and 30 with AAA1 of dynein 1; Table S2. Interacting amino acids with analogues 38, 42, and 45; Table S3. The binding energy of *E* and *Z* isomers of ciliobrevin A and ciliobrevin D; Table S4. Interacting amino acids with dynapyrazole A and B in the protonated and deprotonated states.
