# Supplementary material for: Analysis of the Structural Mechanism of ATP Inhibition at the AAA1 Subunit of Cytoplasmic Dynein-1 Using a Chemical “Toolkit”
Source: Int J Mol Sci. 2021 Jul 19;22(14):7704. doi: 10.3390/ijms22147704 (PMC8304172; doi:10.3390/ijms22147704)
Supplement: Supplementary file 1 [file ijms-22-07704-s001.zip › ijms-1240017-supplementary.pdf]

Supplementary Information

Analysis of the Structural Mechanism of ATP Inhibition at the AAA1 Subunit of Cytoplasmic Dynein-1 Using a Chemical "Toolkit"

Sayi'Mone Tati, Laleh Alisaraie *

School of Pharmacy, Memorial University of Newfoundland, 300 Prince Philip Dr, A1B 3V6, St. John's, Newfoundland, Canada

* Corresponding author
Email: laleh.alisaraie@mun.ca

Figure S1

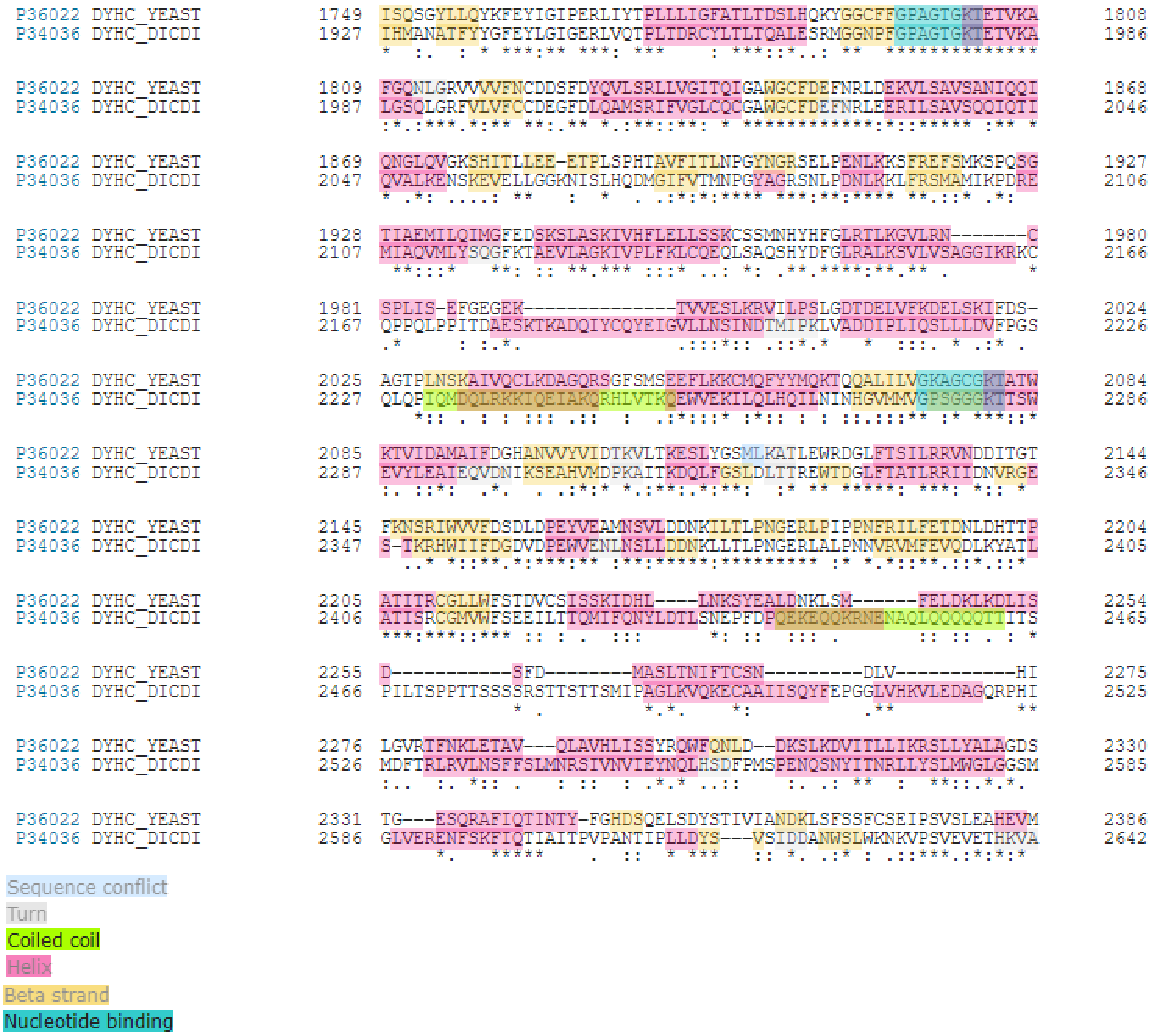

Figure S1: Amino acids sequence alignment of the AAA1 and AAA2 subunits of dynein corresponding to *S. cerevisiae* (1758-2273) and *D. discoideum* (1936-2531) with the ClustalW program. The W-A motif is highlighted in blue. The grey highlights represent sequence similarity. A singular dot (.) indicates that residues belong to a different group of amino acids, while a colon (:) Residues belong to the same group of amino acids and asterisk (*) indicates sequence identity.

Figure S2

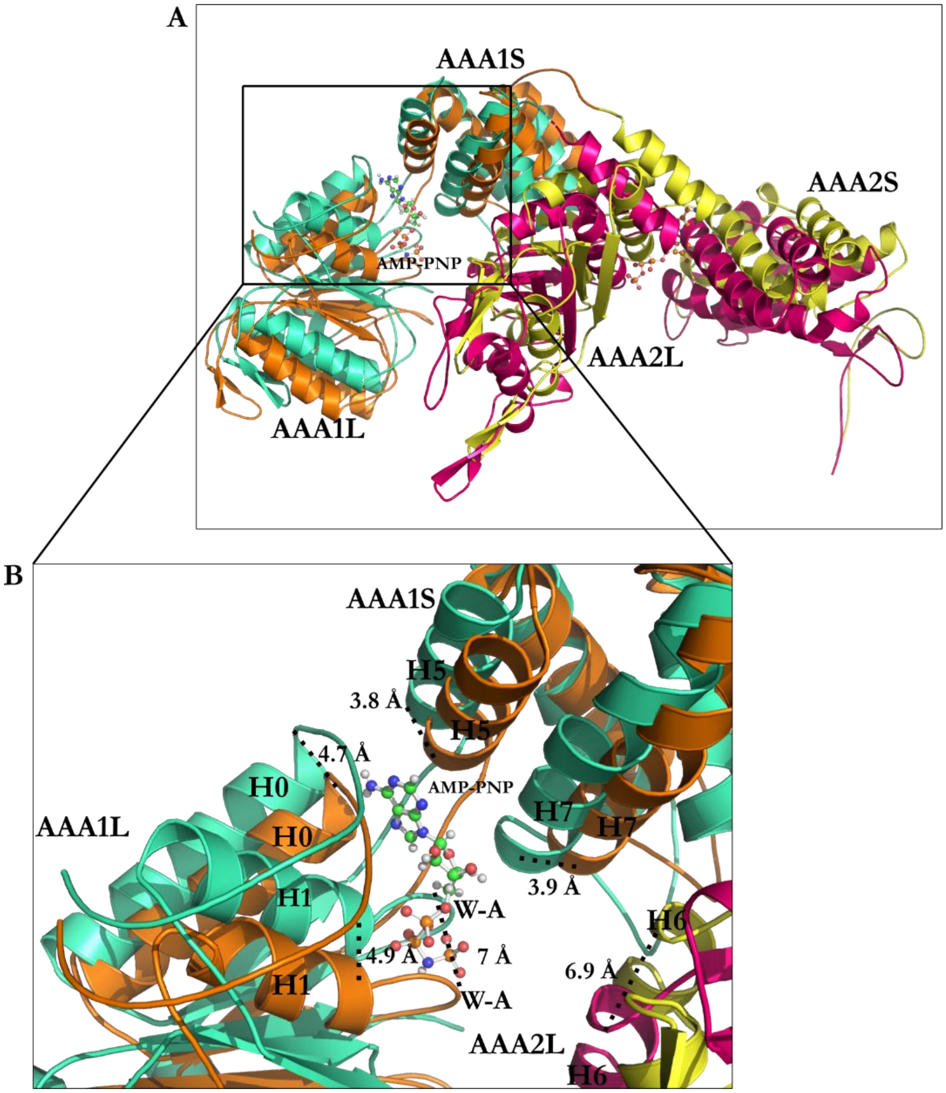

Figure S2: A) Structural alignment of the AAA1 and AAA2 of yeast-AMPPNP (4W8F) and yeast-apo (4AKG), **B)** A close-up of the AAA1 binding site. Helices (H0, H1, H5, and H7) of the AAA1 subdomain, the Walker-A (W-A) loops with the AMP-PNP in the ball and stick representation in green located at the AAA1 nucleotide-binding site and the H6 of the AAA2 subdomains of the yeast-AMPPNP (4W8F) and yeast-apo (4AKG). Color code: yeast-AMPPNP (AAA1 in green cyan and AAA2 in sharp pink) and yeast-apo (AAA1 in orange and AAA2 in yellow)

Figure S3

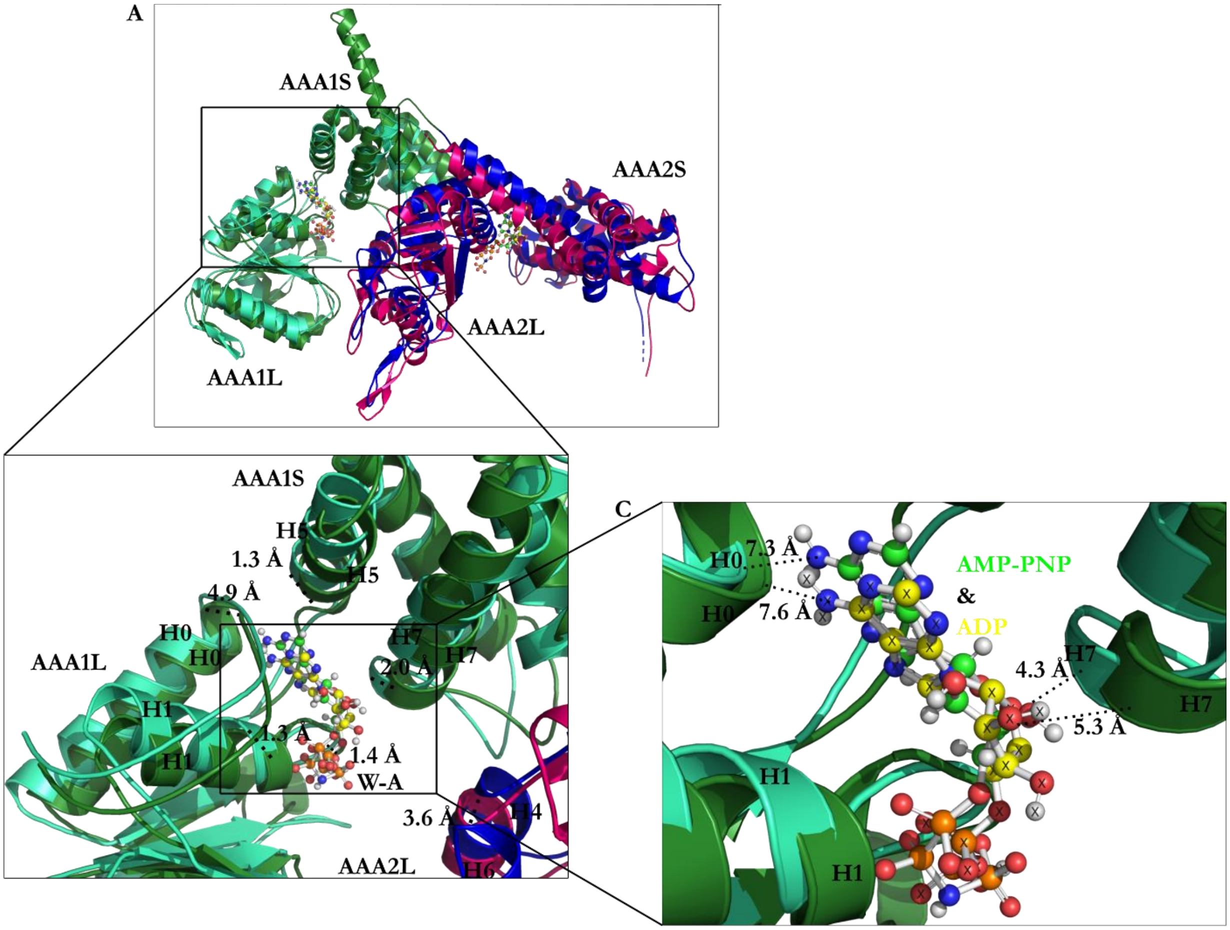

Figure S3: A) Structural alignment of the AAA1 and AAA2 subunit of the *Dictyostelium-ADP* (3VKG) and yeast-AMPPNP (4W8F), **B)** A close-up of the AAA1 binding site. Helices (H0, H1, H5, and H7) of the AAA1 subdomain, the Walker-A (W-A) loops with the AMP-PNP in the ball and stick representation in green and ADP in the ball and stick representation in yellow located at the AAA1 nucleotide-binding site and the H4 of the AAA2 subdomain of the *Dictyostelium-ADP* (3VKG) and the H6 of the AAA2 subdomain of the yeast-apo (4AKG), **C)** Structural alignment of ligands at the AAA1 nucleotide-binding site of the *Dictyostelium-ADP* (3VKG) and yeast-AMPPNP (4W8F) with AMP-PNP in the ball and stick representation in green and ADP in the ball and stick representation in yellow with a cross (x) on its atoms to distinguish them from the atoms of AMP-PNP. Color code: *Dictyostelium*-ADP (AAA1 in green and AAA2 in blue) and yeast-AMPPNP (AAA1 in green cyan and AAA2 in sharp pink)

Figure S4

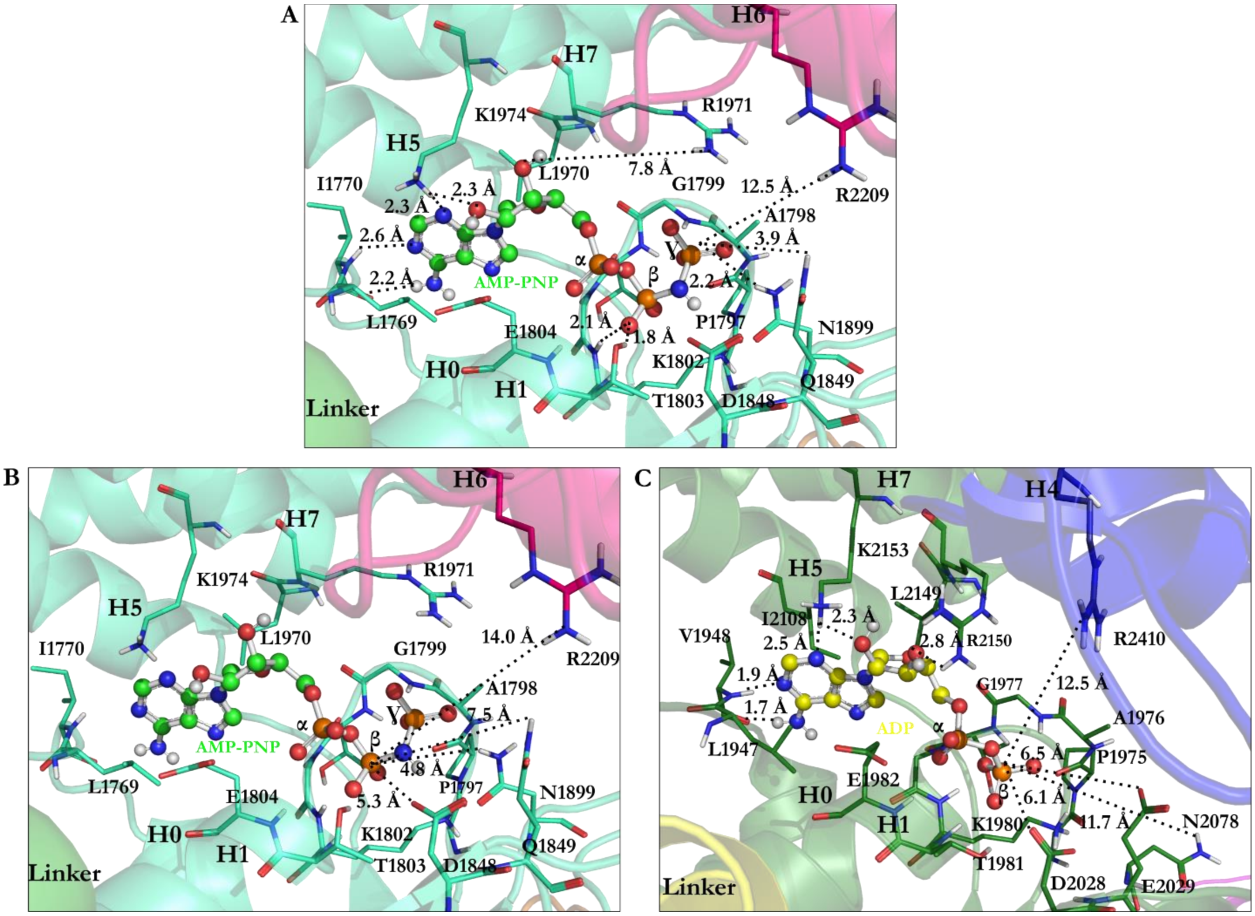

Figure S4: A) AMP-PNP interacting and residues of the AAA1 nucleotide-binding site. Distances between catalytic residues of the AAA1 nucleotide-binding site of dynein and the β-phosphate of **B)** AMP-PNP and **C)** ADP.

Figure S5

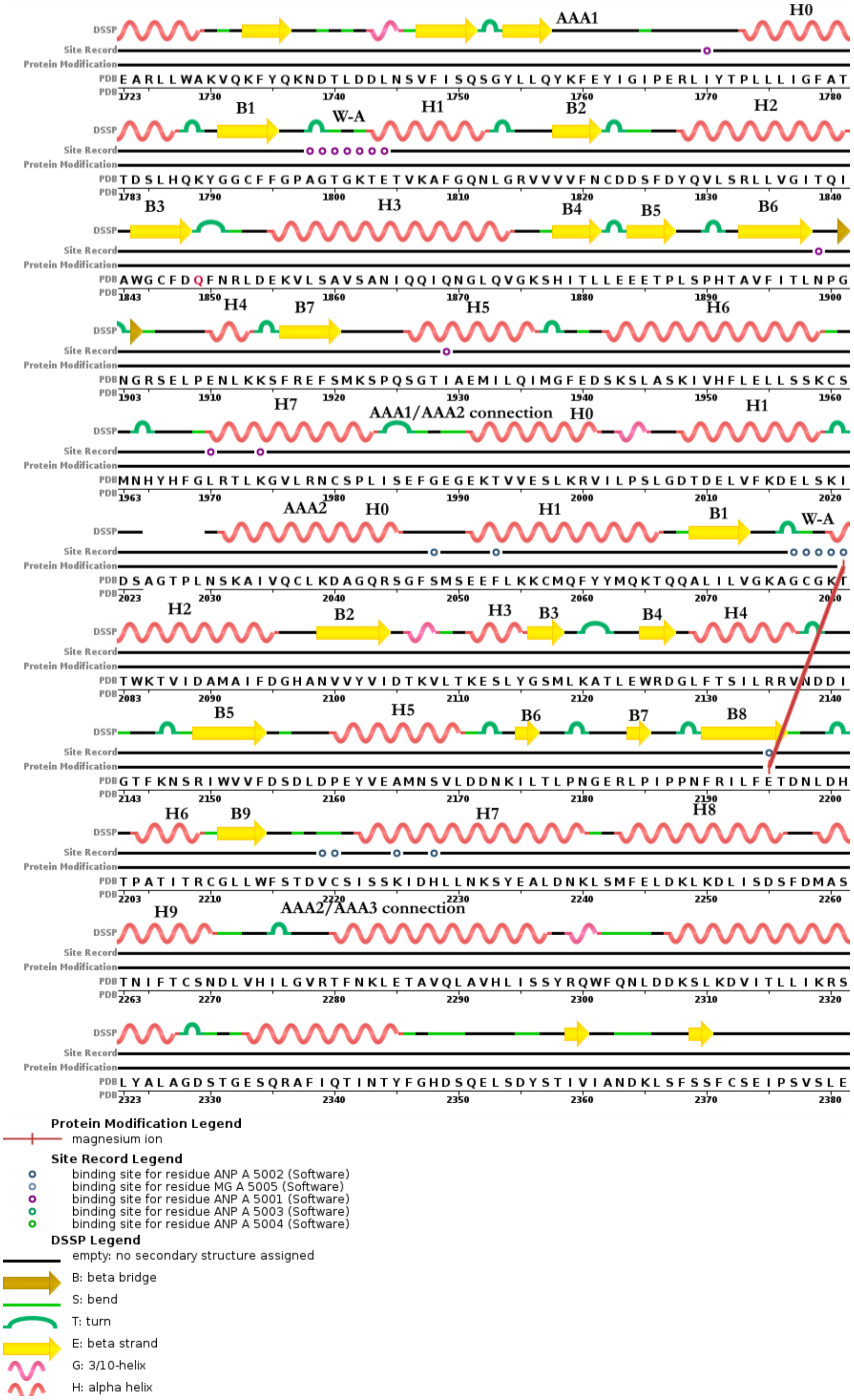

Figure S5: Annotation of the secondary structure of AAA1 and AAA2 domains of cytoplasmic dynein according to the 4W8F crystal structure retrieved from the PDB.

Figure S6

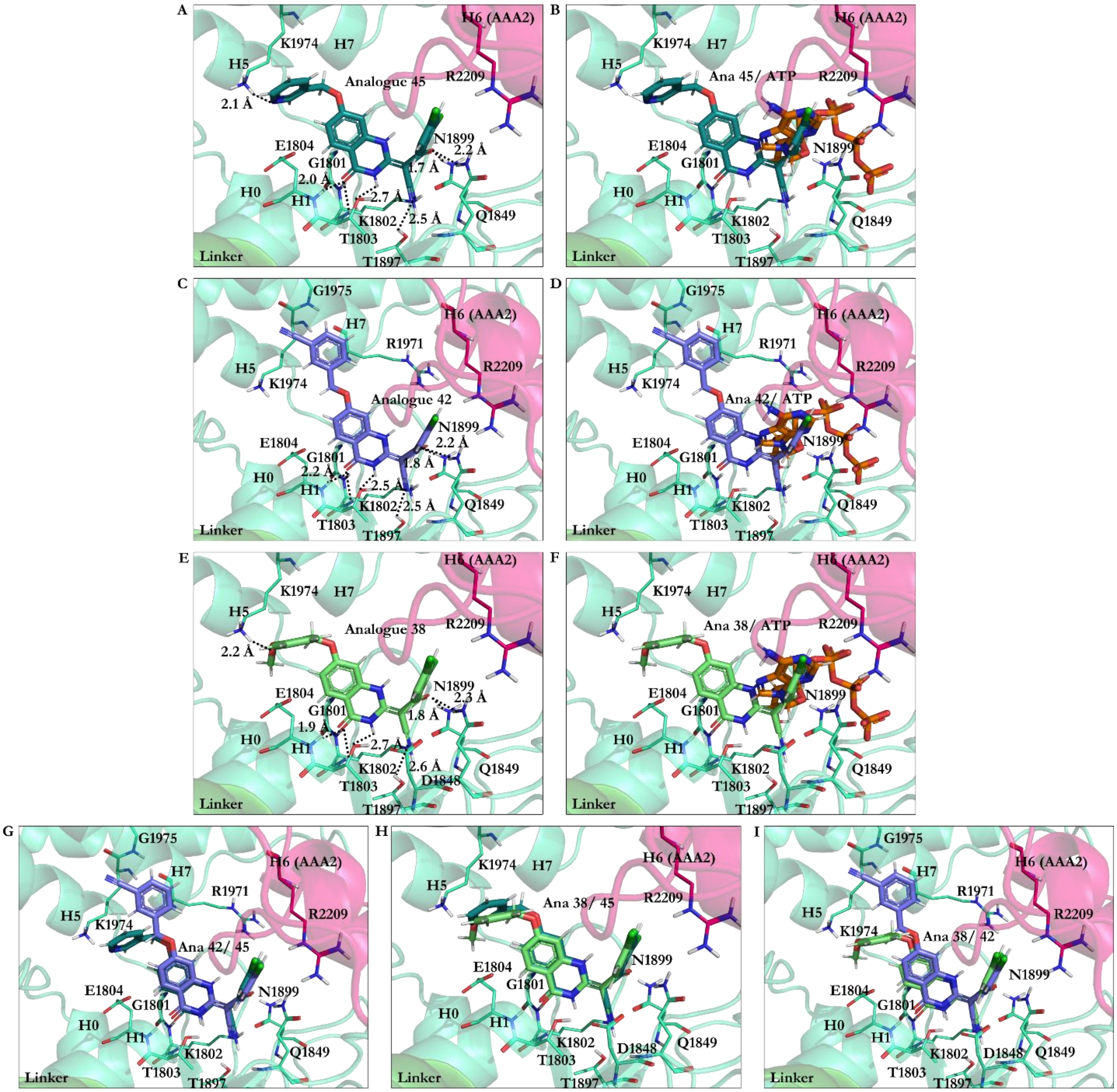

Figure S6: Binding modes of ciliobrevin analogs at the AAA1 binding site of dynein 1. **A)** Analog 45. **B)** Analog 45 superimposed on ATP, **C)** Analog 42, **D)** Analog 42 superimposed on ATP, **E)** Analog 38, **F)** Analog 38 superimposed on ATP. Superimposition of **G)** Analogs 42 and 45, **H)** Analogs 38 and 45, **I)** Analogs 38 and 42.

Figure S7

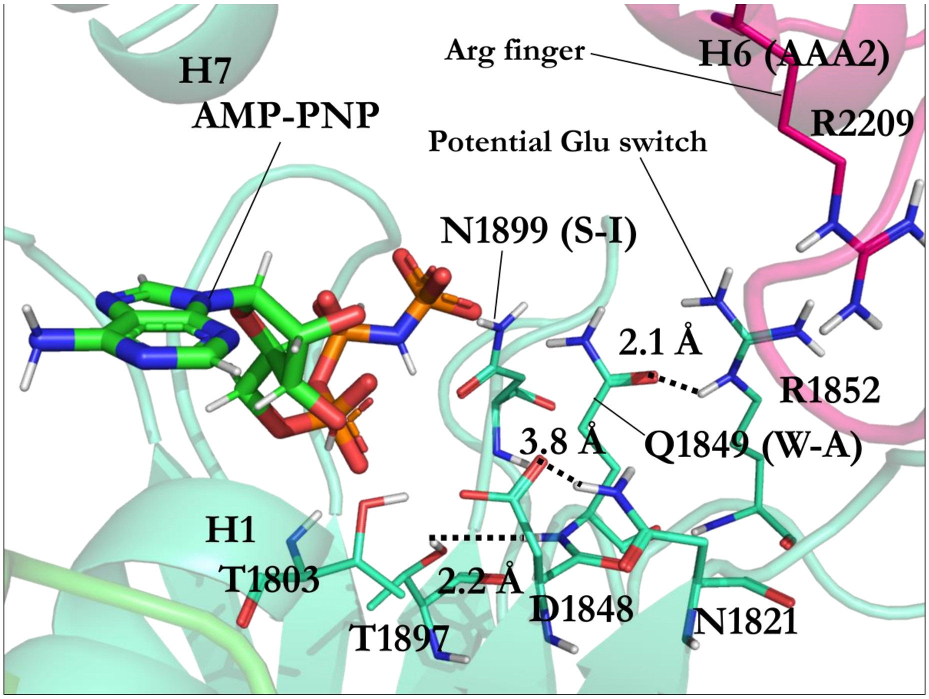

Figure S7: Intramolecular interactions between the catalytic residues at the AAA1 binding site and Arg1852 involving a potential glutamate switch. The docking solution of AMP-PNP is represented in green and stick. Thr1803 and Asp1848 accommodate Mg^{2+} during ATP hydrolysis. Sensor I (N1899), W-B (E1849Q), R-finger (R2209) play catalytic roles during ATP hydrolysis.

Figure S8

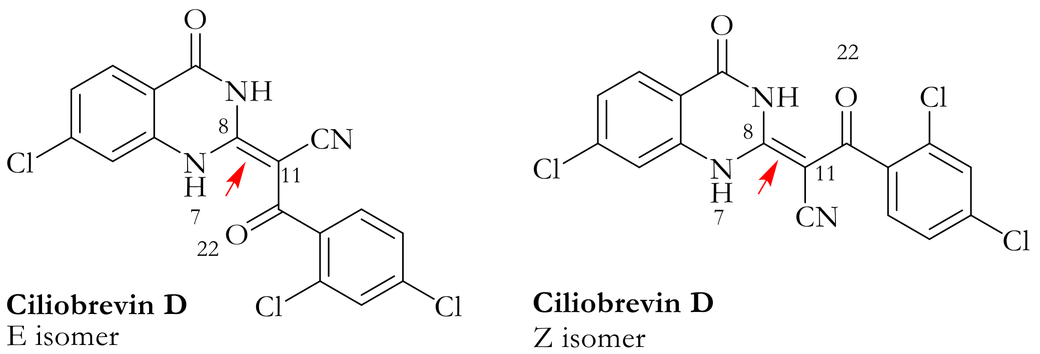

Figure S8: *E* and *Z* configurations of ciliobrevin D. Red arrows point to the double bond between the C8 and C11 atoms.

Figure S9

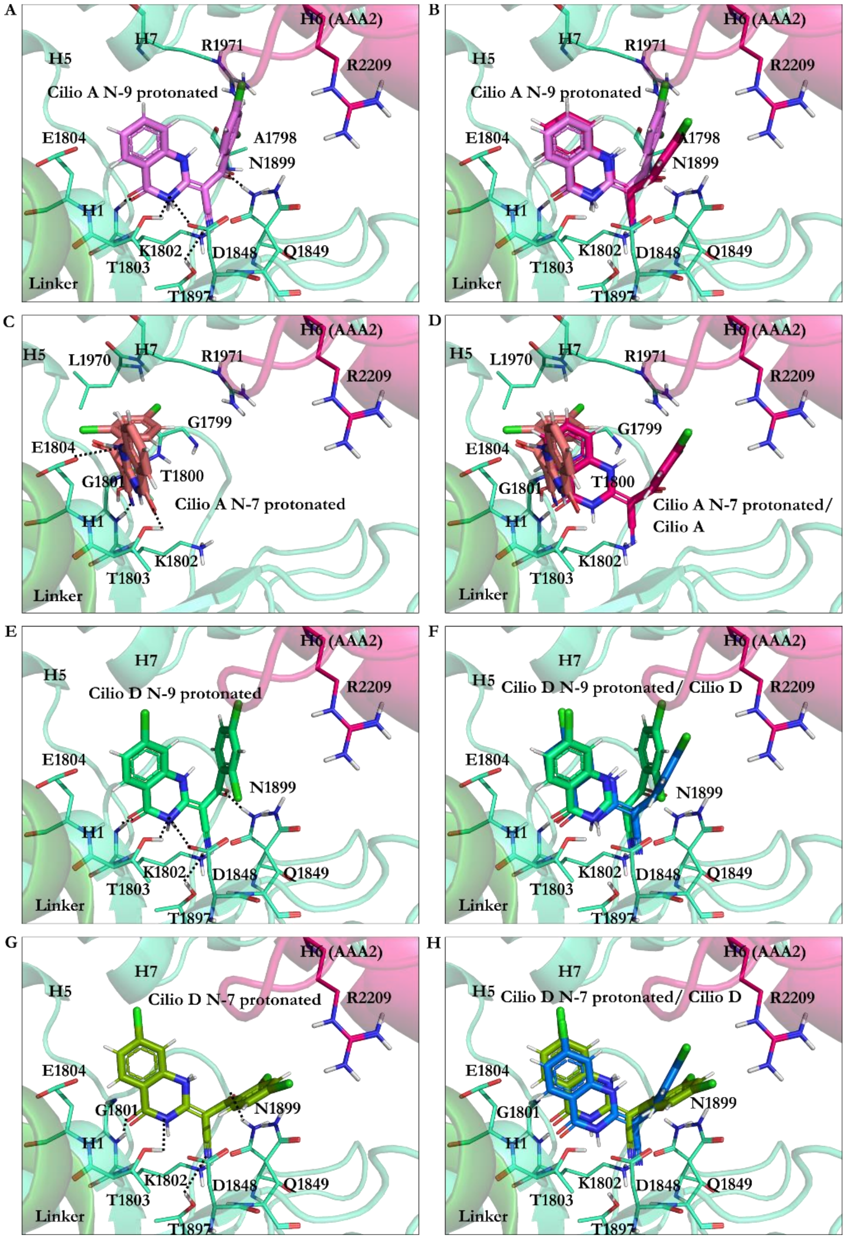

Figure S9: Binding modes of the protonated forms of ciliobrevin at the AAA1 binding site of dynein 1. **A)** Ciliobrevin A protonated at the N9 atom, **B)** Ciliobrevin A protonated at the N9 atom superimposed on ciliobrevin A, **C)** Ciliobrevin A protonated at the N7 atom, **D)** Ciliobrevin A protonated at the N7 atom superimposed on ciliobrevin A, **E)** Ciliobrevin D protonated at the N9 atom, **F)** Ciliobrevin D protonated at the N9 atom superimposed on ciliobrevin D, **G)** Ciliobrevin D protonated at the N7 atom, **H)** Ciliobrevin D protonated at the N7 atom superimposed on ciliobrevin D.

Figure S10

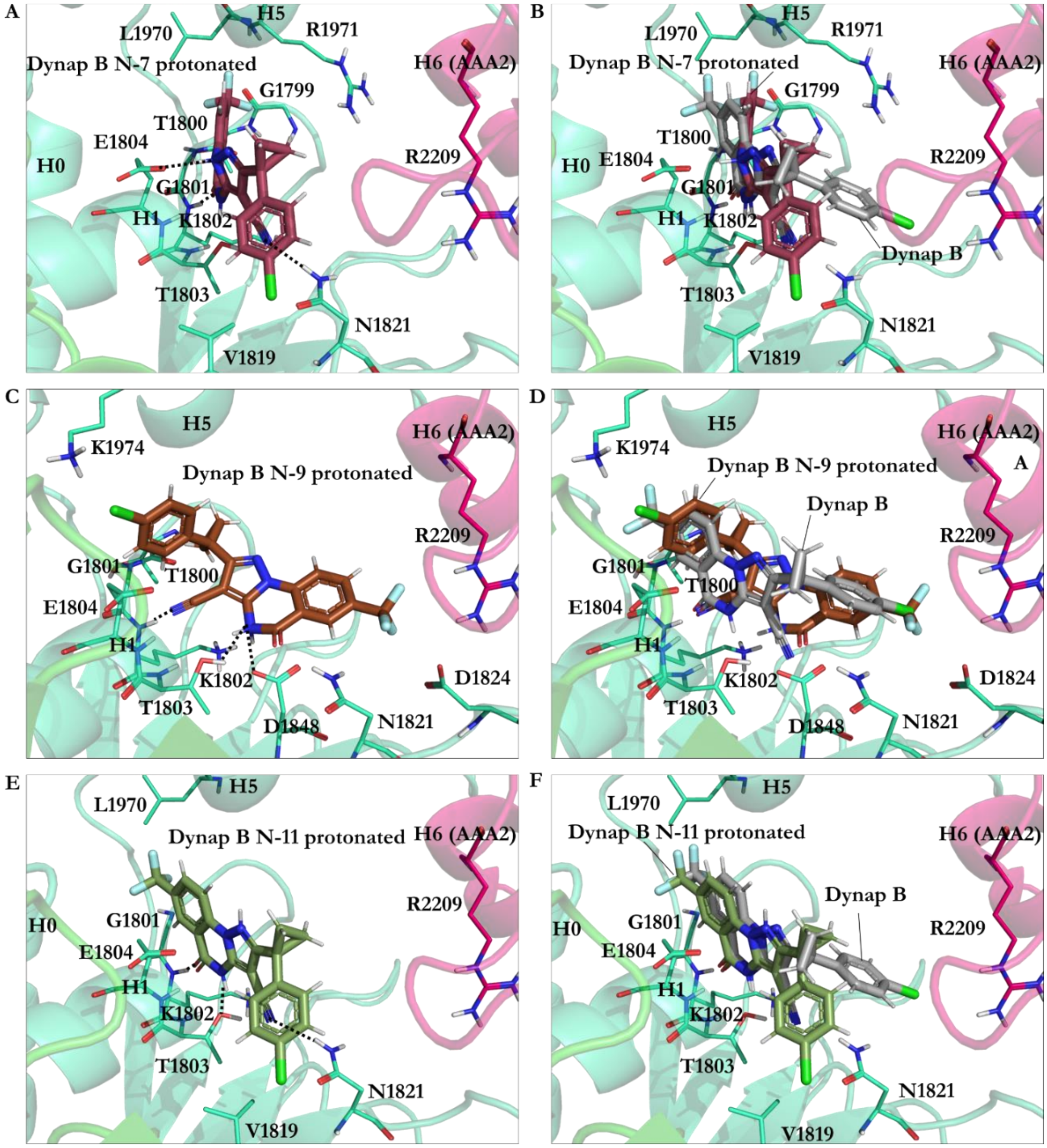

Figure S10: Main binding interactions of the protonated forms of dynapyrazole B at the AAA1 binding site of dynein 1. **A)** Dynapyrazole B protonated at the N7 atom, **B)** Dynapyrazole A protonated at the N7 atom superimposed on dynapyrazole B, **C)** Dynapyrazole B protonated at the N9 atom, **D)** Dynapyrazole B protonated at the N9 atom superimposed on dynapyrazole A, **E)** Dynapyrazole B protonated at the N11 atom, **F)** Dynapyrazole B protonated at the N11 atom superimposed on dynapyrazole B.

Table S1

Compound	Binding Energy (kJ/mol)	Residues interacting with the compound
**Analog 30**	−27.87	Gly1801, Lys 1802, Thr1803, Glu1804, Asp1848, Gln1849, Thr1897, Asn1899
**Analog 29**	−27.37	Gly1801, Lys1802, Thr1803, Glu1804, Asp1848, Gln1849, Thr1897, Asn1899
**Analog 28**	−27.27	Gly1801, Lys1802, Thr1803, Glu1804, Asp1848, Gln1849, Thr1897, Asn1899

Table S1: Networking interactions of analogs 28, 29, and 30 with AAA1 of dynein 1.

Table S2

Compound	Binding energy (kJ/mol)	Residues interacting with the ligand
**Analog 45**	−29.72	Gly1801, Lys1802, Thr1803, Glu1804, Gln1849, Thr1897, Asn1899, Lys1974
**Analog 42**	−27.43	Gly1801, Lys1802, Thr1803, Glu1804, Gln1849, Thr1897, Asn1899, Lys1974, Gly1975, Arg1971
**Analog 38**	−27.27	Gly1801, Lys1802, Thr1803, Glu1804, Asp1848, Gln1849, Thr1897, Asn1899, Lys1974

Table S2: Interacting amino acids with analogs 38, 42, and 45.

Table S3

Compound	Binding energy (kJ/mol)	Residues interacting with the docked ligand
**Ciliobrevin A *E*-isomer**	−26.23	Gly1801, Lys1802, Thr1803, Glu1804, Asp1848, Gln1849, Thr1897, Asn1899
**Ciliobrevin A *Z*-isomer**	−19.41	Thr1803, Asn1821, Asp1848, Gln1849, Arg 1852, Asn1899, Arg1852, Arg1971
**Ciliobrevin D *E*-isomer**	−23.92	Gly1801, Lys1802, Thr1803, Glu1804, Asp1848, Gln1849, Thr1897, Asn1899
**Ciliobrevin D *Z*-isomer**	−19.78	Gly1799, Thr1800, Gly1801, Lys1802, Thr1803, Glu1804, Asp1848, Gln1849, Leu1970, Arg1971, Lys1974
***E*-Analog 22**	−16.83	Ala1798, Gly1969, Tyr1902, Pro1900, Arg1971, His1967
***Z*-Analog 22**	−17.49	Leu1769, Gly1801, Lys1802, Glu1804, Leu1970, Lys1974

Table S3: The binding energy of *E* and *Z* isomers of ciliobrevin A and ciliobrevin D.

Table S4

Compounds	Binding energy (kJ/mol)	Residues interacting with the compound
**Dynapyrazole B N-7 protonated**	−26.95	Gly1799, Thr1800, Gly1801, Lys1802, Thr1803, Glu 1804, Val1819, Asn1821, Leu1970, Arg1971
**Dynapyrazole A N-7protonated**	−25.99	Gly1801, Lys1802, Thr1803, Glu1804, Asn1821
**Dynapyrazole A N-9 protonated**	−19.81	Glu1586, Lys1696, Glu1699, Pro1766, Glu1767, Arg1978
**Dynapyrazole B**	−18.49	Thr1800, Gly1801, Lys1802, Thr1803, Glu1804, Asn1821, Leu1970
**Dynapyrazole B N-9 protonated**	−18.47	Thr1800, Gly1801, Lys1802, Thr1803, Glu1804, Asn1821, Asp1824, Asp1848, Lys1974
**Dynapyrazole A**	−18.17	Gly1801, Lys1802, Thr1803, Glu1804, Asn1821
**Dynapyrazole A N-11 protonated**	−17.49	Lys 1802, Thr1803, Glu1804, Asp1848, Gln1849, Asn1899
**Dynapyrazole B N-11 protonated**	−17.13	Gly1801, Lys1802, Thr1803, Glu1804, Val1819, Asn1821, Leu1970

Table S4: Interacting amino acids with dynapyrazole A and B in the protonated and deprotonated states.
